# Steroidal[17,16-*d*]pyrimidines derived from dehydroepiandrosterone: A convenient synthesis, antiproliferation activity, structure-activity relationships, and role of heterocyclic moiety

**DOI:** 10.1038/srep44439

**Published:** 2017-03-14

**Authors:** Shaoyong Ke, Liqiao Shi, Zhigang Zhang, Ziwen Yang

**Affiliations:** 1 National Biopesticide Engineering Technology Research Center, Hubei Biopesticide Engineering Research Center, Hubei Academy of Agricultural Sciences, Wuhan 430064, People’s Republic of China

## Abstract

A series of steroidal[17,16-*d*]pyrimidines derived from dehydroepiandrosterone were designed and prepared by a convenient heterocyclization reaction. The *in vitro* anticancer activities for these obtained compounds were evaluated against human cancer cell lines (HepG2, Huh-7, and SGC-7901), which demonstrated that some of these heterocyclic pyrimidine derivatives exhibited significantly good cytotoxic activities against all tested cell lines compared with 5-fluorouracil (5-FU), especially, compound **3b** exhibited high potential growth inhibitory activities against all tested cell lines with the IC_50_ values of 5.41 ± 1.34, 5.65 ± 1.02 and 10.64 ± 1.49 μM, respectively, which might be used as promising lead scaffold for discovery of novel anticancer agents.

With the constant increase in cancer mortality, cancer has gradually become one of the most complicated diseases that threaten public health of humans^1^. Although many effective therapeutic methods have been approved for cancer control, chemotherapy still remains a mainstay options for cancer treatment^2^,^3^. However, the emergence of side effects and multidrug-resistance have encouraged us to discover and identify novel synthetic or naturally occurring small molecules with highly effective bioactivity or therapeutic use. Therefore, searching for and developing new chemical entities with special characteristics as effective anticancer molecules are an important endeavor in the field of medicinal chemistry.

It is well known that pyrimidine core has wide occurrence in nature^4^, and which has also represent a typical class of heterocyclic scaffold in various compounds applied in the field of medicinal chemistry^5^,^6^,^7^,^8^,^9^,^10^,^11^,^12^,^13^,^14^,^15^, agrochemicals^16^,^17^,^18^,^19^,^20^,^21^,^22^,^23^,^24^, and materials^25^,^26^,^27^,^28^,^29^. Up to now, many pyrimidines analogues have been demonstrated to exhibit wide pharmacological activities mainly including anticancer, antiviral, antibacterial, antimalarial, antituberculosis activities. In particular, some compounds containing pyrimidine unit have been developed as highly effective anticancer or antibacterial drugs (Fig. 1), which further confirm pyrimidine ring is an important pharmacophore in the discovery of novel active molecules. Meanwhile, natural steroids, particularly dehydroepiandrosterone (DHEA) attract extensive interest of researchers, and many steroidal compounds bearing DHEA moiety have also emerged as highly potential pharmaceutical molecules due to their inherent bioactivity^30^,^31^,^32^. Very recently, a series of DHEA-dihydrazone derivatives have also been investigated by us^33^,^34^, and the results indicated some of the prepared molecules could inhibit the growth of human tumor cell lines, which also identify that this natural steroidal scaffold might contribute to the potential cytotoxic activity.

Due to the above description and our ongoing interest in the discovery of novel functional heterocycles, we wish to integrate the structural features of pyrimidines and DHEA scaffold to a core structure as shown in Fig. 2, and explore the potential antiproliferative effects for these novel molecules derived from natural steroids. So we focused on the convenient synthesis, biological evaluation, and structure-activity relatioships of heterocyclic steroidal analogues. Therefore, a series of steroidal[17,16-*d*]pyrimidines derivatives **3a-p** were synthesized according to the method shown in Fig. 3, and their cytotoxic effects on tumor cell lines (HepG2, Huh-7, SGC-7901) were also investigated by MTT colorimetric method, and the possible structure-activity relationships have also been summarized and discussed. These findings can provide interesting information for discovery of potential chemotherapeutic agents.

## Results and Discussion

### Chemistry

**Figure 3:**
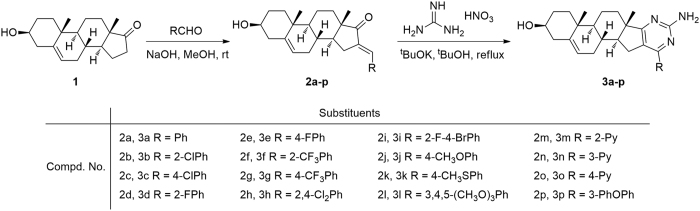
Synthesis of steroidal[17,16-*d*]pyrimidines derivatives.

In this study, a series of heterocyclic steroidal[17,16-*d*]pyrimidines were synthesized via a sequence convenient transformation. The synthetic route for the preparation of these steroidal[17,16-*d*]pyrimidines derivatives **3a-p** is outlined in Fig. 3.

As shown in Fig. 3, the easily available natural steroid DHEA **1** was used as raw materials, which could be readily transferred to various substituted benzylidene-dehydroepiandrosterone derivatives **2a-p** by a simple condensation reaction with various aldehydes in the presence of base. Then the treatment of intermediate **2a-p** with guanidine nitrate formed the steroidal[17,16-*d*]pyrimidines **3a-p** under the condition of base catalysis. Especially, the products for all these two steps are easy to separate and column chromatography isn’t required. All the newly prepared steroidal[17,16-*d*]pyrimidines derivatives **3a-p** and the intermediates **2a-p** gave satisfactory analyses mainly including ^1^H NMR, ^13^C NMR and ESI-MS spectrum, and their chemical structures and basic physiochemical properties were summarized in the Supporting Information.

### Spectroscopy studies

**Figure 4:**
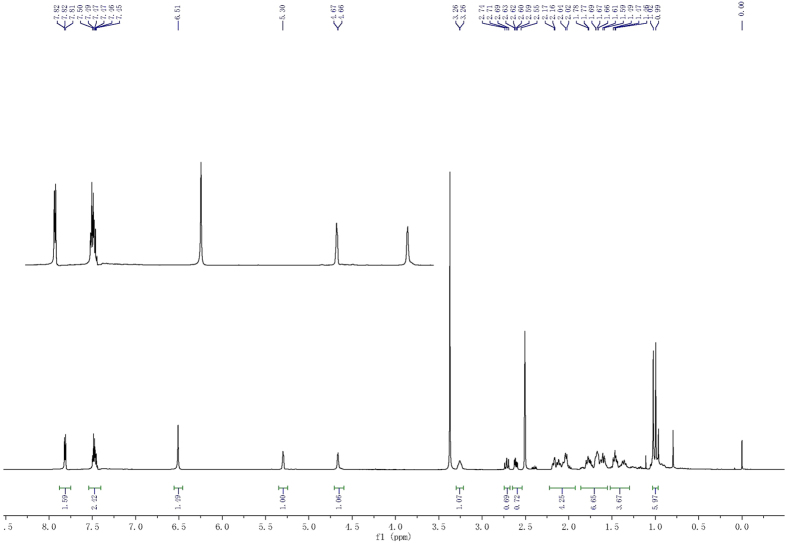
Representative ^1^H NMR spectra for compound **3a**.

All structures of target molecules **3a-p** were determined by their ^1^H NMR, ^13^C NMR and mass spectroscopy, and all these spectral data were in good agreement with their structures. For ^1^H NMR spectrum, all assignments of the signals are based on their chemical shifts and intensity patterns. As shown in the representative ^1^H NMR spectrum (Fig. 4), all ^1^H NMR spectra of compounds **3a-p** revealed the distinctive signals of methine proton attached to hydroxyl group, which showed a multiplet or broad singlet at 3.20–3.57 ppm. The signal for protons of alkene bond in DHEA scaffold was resonated as a singlet or doublet between *δ* 5.19–5.38 ppm. The other set of signals that emerged in their ^1^H NMR spectrum in the range of 3.19–0.85 ppm were assigned to the protons of DHEA skeleton, and the signals at lower fields in the corresponding ^1^H NMR spectrum were attributed to the aromatic protons as indicated in the general structures in Fig. 3. The ^13^C NMR spectra of compounds **3a-p** display obvious peaks in the alkyl region indicating the presence of the DHEA scaffold. Other peaks appearing at lower fields were assigned to the aromatic and heterocyclic moiety. The electron spray impact mass spectra (ESI-MS) for compounds **3a-p** was measured on a Waters ACQUITY UPLC^®^ H-CLASS PDA (Waters^®^) instrument, and the ion peak or adduct ions of the compounds were explored. According to the experimental results, the ESI-MS of compounds **3a-p** exhibit the obvious molecular peak [M + H]^+^ with high abundance (100%) in the positive ion mode. In addition, all feature peaks present in the ^1^H NMR and ^13^C NMR spectra for target derivatives are described in Supporting Information.

### Inhibitory effects on the proliferation of cancer cells for the synthesized compounds

**Figure 5:**
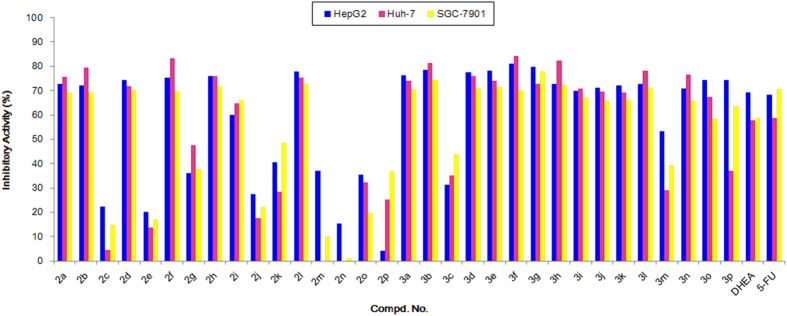
Antitumor activities of compounds **2a-p** and **3a-p** at 40 μg/mL.

All obtained steroidal[17,16-*d*]pyrimidines derivatives **3a-p** and the steroidal intermediates **2a-p** were screened for their potential *in vitro* cytotoxic activity against HepG2 (human hepatocellular liver carcinoma), Huh-7 (human heptoma), and SGC-7901 (human gastric cancer) cell lines by the standard MTT assay^35^ using 5-fluorouracil as a control. The preliminary screened results were depicted in Fig. 5 and Table 1, and the IC_50_ value represents the drug concentration required to inhibit cell growth by 50%.

Generally, as indicated in Fig. 5, most of the heterocyclic steroidal[17,16-*d*]pyrimidines derivatives **3a-p** displayed good cytotoxic activities than the corresponding steroidal intermediates **2a-p**. Notably, the compounds **3a**, **3b**, **3d**, **3e**, **3f**, **3g**, **3h**, and **3l** exhibited excellent inhibitory activities against all three cell lines with 70–82% growth inhibition at the concentration of 40 μg/mL compared to the positive control 5-fluorouracil (58.6–70.5%). From Fig. 5, we also can observe that the typical compounds **3a**, **3b**, **3d**, **3e**, **3f**, **3g**, **3h**, and **3l** also presented better inhibitory activities than the natural compound DHEA (57.9–69.4%), which indicated these heterocyclic steroidal[17,16-*d*]pyrimidines can be used as a potential lead compounds for designing of novel anticancer drugs.

Meanwhile, the bioassay revealed many of these heterocyclic compounds mainly including **3a**, **3b**, **3d**, **3e**, **3f**, **3g**, **3h**, and **3l** had good cytotoxic activities compared to 5-fluorouracil (Fig. 5), so in order to explore the highly potential activities, the IC_50_ values for these compounds **3a-p** were further investigated and compared to **2a-p**, DHEA and 5-fluorouracil. The popential inhibitory activities expressed as IC_50_ values for all compounds are shown in Table 1, which also demonstrated that some of the designed steroidal[17,16-*d*]pyrimidines derivatives **3a-p** exhibited obviously inhibitory activities than the control 5-fluorouracil. Among all the compounds indicated in Table 1, compounds **3a**, **3b**, **3d**, **3e**, **3f**, **3g**, **3h**, **3i**, **3j**, **3k**, and **3l** showed promising cytotoxic activities (Entries 17, 18, and 20–28) against all three cell lines. Especially, we also can find that compounds **3b**, **3g**, **3h**, **3i** and **3l** exhibited a certain selective inhibition (Entries 18, 23, 24, 25 and 28) against all three cancer cell lines than the reference 5-fluorouracil. In addition, compound **3b** showed the highest inhibitory effect on HepG2, Huh-7 and SGC-7901 cell lines, with an IC_50_ values of 5.41 ± 1.34, 5.65 ± 1.02 and 10.64 ± 1.49 μM, respectively.

**Figure 6:**
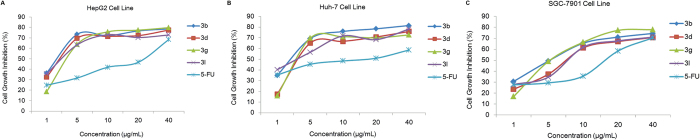
Dose-response analysis of cell growth inhibition activity for the potential compounds **3b**, **3d**, **3g**, **3l** and **5-FU** (positive control) against HepG2 (**A**), Huh-7 (**B**), and SGC-7901 (**C**) cell lines.

Moreover, the dose-response relationship of cell growth inhibition for highly potential compounds **3b**, **3d**, **3g**, **3l** and 5-fluorouracil have been presented in Fig. 6, which indicated that these heterocyclic compounds obviously inhibited HepG2, Huh-7, and SGC-7901 cell proliferation in a concentration-dependent manner. Especially, it should be pointed out that compound **3b** containing an ortho-chlorophenyl unit (Entry 18 in Table 1) exhibited the most promising growth inhibitory effects on all three cell lines with the IC_50_ values of 5.41 ± 1.34, 5.65 ± 1.02 and 10.64 ± 1.49 μM, respectively, which was significantly better than the control 5-fluorouracil and DHEA.

### Structure and activity relationships (SARs)

**Figure 7:**
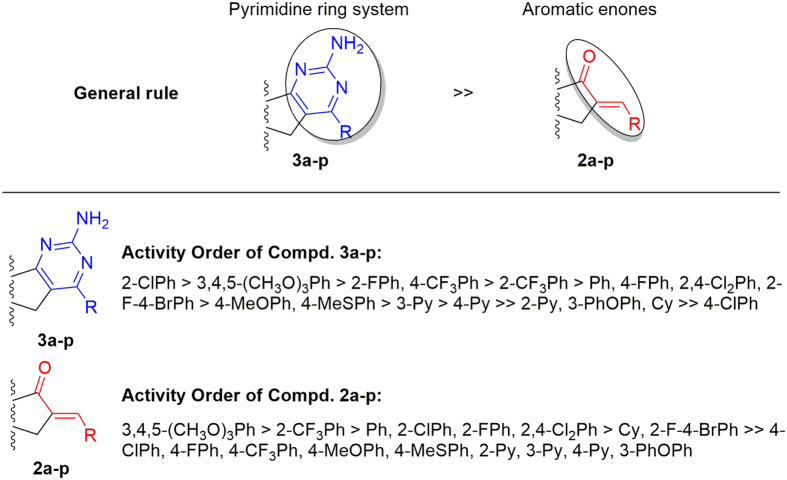
General structure-activity profile for these steroidal derivatives.

The structure evolution here was to modify DHEA scaffold with pyrimidine ring system (**3a-p**) and aromatic enones (**2a-p**), respectively (Fig. 7). According to the *in vitro* bioassay results shown in Fig. 5 and Table 1, the possible structure-activity profile for these prepared steroidal derivatives can be obtained.

As indicated in Fig. 7, the compounds bearing pyrimidine ring system are general present better inhibition activities than the compounds modified by aromatic enones, which proved the importantance of heterocyclic pyrimidines. In addition, for the compounds containing pyrimidine ring system, the compounds bearing 2-ClPh and 3,4,5-(MeO)_3_Ph group present the highest potential activities, however, when the substituents R are 2-Py, 3-Py, 4-Py, and 3-PhOPh, which indicate low efficacy. Also, within the series of pyridine derivatives, the compound **3m** bearing pyridin-2-yl substituent obviously decrease the cytotoxic activity. On the other hand, for the compounds containing aromatic enone moiety, the results testified that compounds containing 3,4,5-(MeO)_3_Ph and 2-CF_3_Ph group exhibited higher activity than the compounds bearing other substituents. From Table 1, we also can find that, within the series of halgen derivatives, it is clear that the ortho-substituted compound is better than the para-substituted compound. In particular, the two compounds containing 3,4,5-(MeO)_3_Ph group (**2l** and **3l**) all present good inhibitory activities in these two systems, which may be due to the steric size of trisubstituted phenyl group is favourable for the binding to receptor. Taking into account these findings, it can be speculated it would have been more interesting to test the ortho-hydroxy and 3,4,5-trihydroxy substituted derivative, and the special properties of hydroxyl group will be helpful to increase the activity. These preliminary structure-activity relationships were to identify the target steroidal[17,16-*d*]pyrimidines derivatives that could serve as potential lead antitumor molecules for drug discovery, and the further structural optimization based on these obtained SAR are well under way in our laboratory.

## Conclusion

Sixteen steroidal[17,16-*d*]pyrimidines derived from dehydroepiandrosterone were designed and synthesized *via* a sequence transformation, and their *in vitro* inhibitory activities against cell proliferation were evaluated. From the present data it was found that some of these heterocyclic steroidal[17,16-*d*]pyrimidines displayed significantly good cytotoxic activities against HepG2, Huh-7 and SGC-7901 cell lines compared to the reference 5-fluorouracil, which might be used as promising lead compounds for discovery of novel antitumor molecules. Further structural optimization and possible mechanism on these steroidal[17,16-*d*]pyrimidines will be investigated in due courses.

## Experimental

### Synthesis of target compounds

The instrumentation, chemicals, synthetic procedures and characterization were provided in supplementary data.

### Biological evaluation

The *in vitro* cytotoxic activities of these steroidal molecules **2a-p** and **3a-p** against several human cancer cell lines (HepG2, Huh-7, SGC-7901) were determined using the MTT [3-(4,5-dimethylthiazol-2-yl)-2,5-diphenyltetrazoliumbromide] method^35^, and the general procedures were previously reported in literatures^36^,^37^. All the experimental data were analyzed by SPSS software, and the 50% inhibitory concentrations (IC_50_) of each molecule for the different cell lines were also measured. All biological evaluation was performed in triplicate on three independent experiments, and measurement data were expressed as the mean ± S. D.

## Additional Information

**How to cite this article:** Ke, S. *et al*. Steroidal[17,16-*d*]pyrimidines derived from dehydroepiandrosterone: A convenient synthesis, antiproliferation activity, structure-activity relationships, and role of heterocyclic moiety. *Sci. Rep.* **7**, 44439; doi: 10.1038/srep44439 (2017).

**Publisher's note:** Springer Nature remains neutral with regard to jurisdictional claims in published maps and institutional affiliations.

## Supplementary Material

Supporting Information

## Figures and Tables

**Figure 1:**
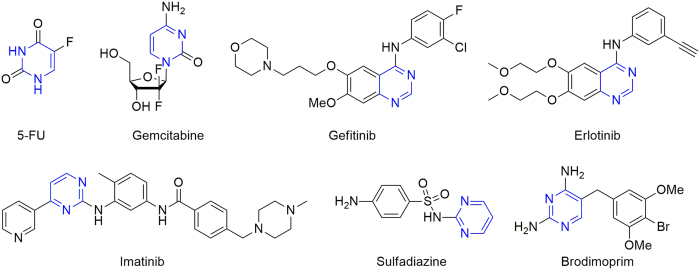
Representative structures for several pyrimidine derivatives.

**Figure 2:**
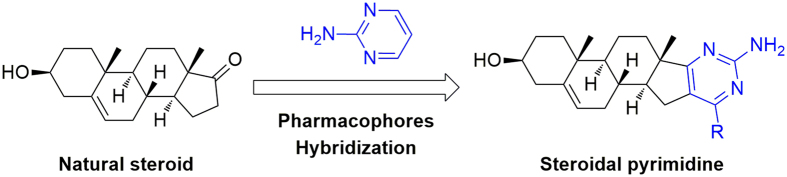
Design strategy of steroidal[17,16-*d*]pyrimidines.

**Table 1:** Cytotoxic activity of the steroidal derivatives.

Entry	Compd.No.	Substrates (R)	*In vitro* cytotoxicity IC_50_^a^ (μM)		
			HepG2^b^	Huh-7^b^	SGC-7901^b^
1	**2a**	Ph	31.74 ± 6.11	29.24 ± 5.66	31.66 ± 7.71
2	**2b**	2-ClPh	29.21 ± 2.78	27.67 ± 3.71	27.04 ± 4.58
3	**2c**	4-ClPh	>95	>95	>95
4	**2d**	2-FPh	35.24 ± 3.96	29.60 ± 7.00	30.04 ± 5.18
5	**2e**	4-FPh	>100	>100	>100
6	**2f**	2-CF_3_Ph	24.09 ± 4.91	18.30 ± 2.14	21.63 ± 3.31
7	**2g**	4-CF_3_Ph	>90	>90	>90
8	**2h**	2,4-Cl_2_Ph	33.50 ± 3.44	27.17 ± 5.72	25.06 ± 5.07
9	**2i**	2-F-4-BrPh	72.21 ± 9.40	50.88 ± 9.64	34.25 ± 6.10
10	**2j**	4-CH_3_OPh	>95	>95	>95
11	**2k**	4-CH_3_SPh	>90	>90	>90
12	**2l**	3,4,5-(CH_3_O)_3_Ph	16.68 ± 2.55	15.93 ± 3.28	18.85 ± 3.43
13	**2m**	2-Py	>100	>100	>100
14	**2n**	3-Py	>100	>100	>100
15	**2o**	4-Py	>100	>100	>100
16	**2p**	3-PhOPh	>85	>85	>85
17	**3a**	Ph	17.94 ± 2.19	12.98 ± 3.25	20.49 ± 3.76
18	**3b**	2-ClPh	5.41 ± 1.34	5.65 ± 1.02	10.64 ± 1.49
19	**3c**	4-ClPh	>85	>85	>85
20	**3d**	2-FPh	9.58 ± 3.60	12.53 ± 2.05	17.33 ± 2.61
21	**3e**	4-FPh	16.80 ± 2.91	21.92 ± 1.96	23.61 ± 3.05
22	**3f**	2-CF_3_Ph	11.18 ± 2.46	13.87 ± 2.26	14.88 ± 1.53
23	**3g**	4-CF_3_Ph	12.17 ± 3.91	7.43 ± 1.39	11.11 ± 0.83
24	**3h**	2,4-Cl_2_Ph	15.34 ± 3.33	6.02 ± 0.25	13.35 ± 3.52
25	**3i**	2-F-4-BrPh	14.77 ± 4.52	8.69 ± 1.21	18.88 ± 3.36
26	**3j**	4-CH_3_OPh	19.60 ± 5.17	20.57 ± 5.75	25.71 ± 2.07
27	**3k**	4-CH_3_SPh	20.42 ± 2.86	22.85 ± 6.42	24.67 ± 4.49
28	**3l**	3,4,5-(CH_3_O)_3_Ph	6.59 ± 1.78	5.38 ± 1.84	15.71 ± 2.34
29	**3m**	2-Py	84.48 ± 6.77	>95	>95
30	**3n**	3-Py	22.84 ± 5.09	25.10 ± 6.87	33.97 ± 6.44
31	**3o**	4-Py	29.86 ± 7.30	35.34 ± 10.09	60.77 ± 2.67
32	**3p**	3-PhOPh	32.33 ± 6.86	>75	40.63 ± 2.23
33	**DHEA**^c^	—	39.04 ± 10.26	>100	>95
34	**5-FU**^d^	—	>100	>95	>100

^a^IC_50_ – Compound concentration required to inhibit tumor cell proliferation by 50%.

^b^Abbreviations: HepG2 – Human hepatocellular liver carcinoma cell line; Huh-7 – Human hepatoma cell line; SGC-7901 – Human gastric cancer cell line.

^c^DHEA – Dehydroepiandrosterone.

^d^5-Fluorouracil, used as a positive control.

## References

[b1] Siegel R., Ma J., Zou Z. & Jemal A. Cancer statistics, 2014. CA Cancer J. Clin. 64, 9–29 (2014). 24399786 10.3322/caac.21208

[b2] Nasr T., Bondock S. & Youns M. Anticancer activity of new coumarin substituted hydrazide-hydrazone derivatives. Eur. J. Med. Chem. 76, 539–548 (2014). 24607878 10.1016/j.ejmech.2014.02.026

[b3] Avin B. R. V. . Synthesis and tumor inhibitory activity of novel coumarin analogs targeting angiogenesis and apoptosis. Eur. J. Med. Chem. 75, 211–221 (2014). 24534537 10.1016/j.ejmech.2014.01.050

[b4] Lagoja I. M. Pyrimidine as constituent of natural biologically active compounds. Chem. Biodiv. 2, 1–50 (2005). 10.1002/cbdv.200490173 17191918

[b5] Carter D. S. . Identification and SAR of novel diaminopyrimidines. Part 1: The discovery of RO-4, a dual P2X_3_/P2X_2/3_ antagonist for the treatment of pain. Bioorg. Med. Chem. Lett. 19, 1628–1631 (2009). 19231180 10.1016/j.bmcl.2009.02.003

[b6] Piotrowski D. W. . Identification of tetrahydropyrido[4,3-*d*]pyrimidine amides as a new class of orally bioavailable TGR5 agonists. ACS Med. Chem. Lett. 4, 63–68 (2013). 24900564 10.1021/ml300277t PMC4027551

[b7] Chen P. . Synthesis, *in vitro* antimicrobial and cytotoxic activities of novel pyrimidine-benzimidazol combinations. Bioorg. Med. Chem. Lett. 24, 2741–2743 (2014). 24798098 10.1016/j.bmcl.2014.04.037

[b8] Zhang Q. . Identification of type II inhibitors targeting BRAF using privileged pharmacophores. Chem. Biol. Drug Des. 83, 27–36 (2014). 24164966 10.1111/cbdd.12198

[b9] Gad H. . MTH1 inhibition eradicates cancer by preventing sanitation of the dNTP pool. Nature 508, 215–221 (2014). 24695224 10.1038/nature13181

[b10] Yu X., Shi L. & Ke S. Acylhydrazone derivatives as potential anticancer agents: Synthesis, bio-evaluation and mechanism of action. Bioorg. Med. Chem. Lett. 25, 5772–5776 (2015). 26546214 10.1016/j.bmcl.2015.10.069

[b11] Su T. . Discovenry of novel PDE9 inhibitors capable of inhibiting Aβ aggregation as potential candidates for the treatment of Alzheimer’s disease. Sci. Rep. 6, 21826 (2016). 26911795 10.1038/srep21826 PMC4766439

[b12] Venugopala K. N. . Design, synthesis, and computational studies on dihydropyrimidine scaffolds as potential lipoxygenase inhibitors and cancer chemopreventive agents. Drug Des. Dev. Ther. 9, 911–921 (2015). 10.2147/DDDT.S73890 PMC4338777 25733811

[b13] Nagarajan S. . An eco-friendly and water mediated product selective synthesis of 2-aminopyrimidines and their *in vitro* anti-bacterial evaluation. Bioorg. Med. Chem. Lett. 24, 4999–5007 (2014). 25280779 10.1016/j.bmcl.2014.09.027

[b14] Ma W. . One-pot synthesis and antiproliferative activity of novel 2,4-diaminopyrimidine derivatives bearing piperidine and piperazine moieties. Eur. J. Med. Chem. 84, 127–134 (2014). 25016234 10.1016/j.ejmech.2014.07.017

[b15] Buron F., Mérour J. Y., Akssira M., Guillaumet G. & Routier S. Recent advances in the chemistry and biology of pyridopyrimidines. Eur. J. Med. Chem. 95, 76–95 (2015). 25794791 10.1016/j.ejmech.2015.03.029

[b16] Xu Y., Yang J., Ren L., Hao S. & Liu C. Research progress on pyrimidine compounds with insecticidal activities. Agrochemicals 50, 474–478 (2011).

[b17] Wu Q., Song B., Jin L. & Hu D. Research advances in synthesis and antifungal activity of pyrimidine compounds. Chin. J. Org. Chem. 29, 365–379 (2009).

[b18] Azab M. E., Roussef M. M. & El-Bordany E. A. Synthesis and antibacterial evaluation of novel heterocyclic compounds containing a sulfonamido moiety. Molecules 18, 832–844 (2013). 23344196 10.3390/molecules18010832 PMC6270435

[b19] Zheng Z., Chen J., Liu G., Li Y. & Li Z. Syntheis and herbicidal activity of novel 4-substituted pyrimidinyl-phenyl-sulfonylureas. Chin. J. Pestic. Sci. 14, 607–611 (2012).

[b20] Liu X., Zhang L., Tan J. & Xu H. Design and synthesis of N-alkyl-N’-substituted 2,4-dioxo-3,4-dihydropyrimidin-1-diacylhydrazine derivatives as ecdysone receptor agonist. Bioorg. Med. Chem. 21, 4687–4697 (2013). 23757207 10.1016/j.bmc.2013.05.010

[b21] Ma H., Zhang J., Xia X., Kang J. & Li J. Design, synthesis and herbicidal evaluation of novel 4-(1*H*-pyrazol-1-yl)pyrimidine derivatives. Pest Manag. Sci. 71, 1189–1196 (2015). 25256846 10.1002/ps.3918

[b22] Wang M., Sun L., Wan F. & Jiang L. Synthesis and phytotoxic activity of novel acylthiourea and 2*H*-1,2,4-thiadiazolo[2,3-*α*]pyrimidine derivatives. J. Pestic. Sci. 37, 15–19 (2012).

[b23] Li Y. Syntheris and herbicidal activity of *N*-[2-(4,6-dimethoxypyrimidin-2-yloxy)benzylidene]substituted amine derivatives. Chin. J. Pestic. Sci. 13, 645–648 (2011).

[b24] Zhao L. . Design, synthesis and antifungal activity against *Valsa Mali* of the triamino substitued triazines bearing aminopyrimidine group. Chem. J. Chin. Univ. 32, 2795–2799 (2011).

[b25] Liu B. . Novel pyrimidine-based amphiphilic molecules: synthesis, spectroscopic properties and applications in two-photon fluorescence microscopic imaging. J. Mater. Chem. 17, 2921–2929 (2007).

[b26] Zonouzi A., Hosseinzadeh F., Karimi N., Mirzazadeh R. & Ng S. W. Novel approaches for the synthesis of a library of fluorescent chromenopyrimidine derivatives. ACS Comb. Sci. 15, 240–246 (2013). 23547948 10.1021/co300141j

[b27] Xue W., Li L., Li Q. & Wu A. Novel furo[2,3-*d*] pyrimidine derivative as fluorescent chemosensor for HSO_4_^−^. Talanta 88, 734–738 (2012). 22265567 10.1016/j.talanta.2011.11.033

[b28] Kubota Y., Ozaki Y., Funabiki K. & Matsui M. Synthesis and fluorescence properties of pyrimidine mono- and bisboron complexes. J. Org. Chem. 78, 7058–7067 (2013). 23829606 10.1021/jo400879g

[b29] Mati S. S., Chall S., Konar S., Rakshit S. & Bhattacharya S. C. Pyrimidine-based fluorescent zinc sensor: Photo-physical characteristics, quantum chemical interpretation and application in real samples. Sensor Actuat. B: Chem. 201, 204–212 (2014).

[b30] Lednicer D. Steroid Chemistry at a Glance, John Wiley & Sons Ltd.(2011).

[b31] El Kihel L. Oxidative metabolism of dehydroepiandrosterone (DHEA) and biologically active oxygenated metabolites of DHEA and epiandrosterone (EpiA) – Recent reports. Steroids 77, 10–26 (2012). 22037250 10.1016/j.steroids.2011.09.008

[b32] Stárka L., Dušková M. & Hill M. Dehydroepiandrosterone: A neuroactive steroid. J. Steroid Biochem. Mol. Biol. 145, 254–260 (2015). 24704258 10.1016/j.jsbmb.2014.03.008

[b33] Ke S., Wei Y., Shi L., Yang Q. & Yang Z. Synthesis of novel steroid derivatives derived from dehydroepiandrosterone as potential anticancer agents. Anti-Cancer Agents Med. Chem. 13, 1291–1298 (2013). 10.2174/18715206113139990323 23547874

[b34] Ke S., Shi L. & Yang Z. Discovery of novel isatin–dehydroepiandrosterone conjugates as potential anticancer agents. Bioorg. Med. Chem. Lett. 25, 4628–4631 (2015). 26320625 10.1016/j.bmcl.2015.08.041

[b35] Alley M. C. . Feasibility of drug screening with panels of human tumor cell lines using a microculture tetrazolium assay. Cancer Res. 48, 589–601 (1988). 3335022

[b36] Ke S. . Heterocycle-functional gramine analogues: Solvent- and catalyst-free synthesis and their inhibition activities against cell proliferation. Eur. J. Med. Chem. 54, 248–254 (2012). 22647218 10.1016/j.ejmech.2012.05.003

[b37] Shi L. . Anthranilic acid-based diamides derivatives incorporating aryl-isoxazoline pharmacophore as potential anticancer agents: Design, synthesis and biological evaluation. Eur. J. Med. Chem. 54, 549–556 (2012). 22727445 10.1016/j.ejmech.2012.06.001

