# Supplementary material for: Steroidal[17,16-d]pyrimidines derived from dehydroepiandrosterone: A convenient synthesis, antiproliferation activity, structure-activity relationships, and role of heterocyclic moiety
Source: Sci Rep. 2017 Mar 14;7:44439. doi: 10.1038/srep44439 (PMC5349525; doi:10.1038/srep44439)

Steroidal[17,16-*d*]pyrimidines derived from dehydroepiandrosterone: A convenient synthesis, antiproliferation activity, structure-activity relationships, and role of heterocyclic moiety

Shaoyong Ke, Liqiao Shi, Zhigang Zhang, and Ziwen Yang

National Biopesticide Engineering Technology Research Center, Hubei Biopesticide Engineering Research Center, Hubei Academy of Agricultural Sciences, Wuhan 430064, People's Republic of China

Address for correspondence: Shaoyong Ke, National Biopesticide Engineering Technology Research Center, Hubei Biopesticide Engineering Research Center, Hubei Academy of Agricultural Sciences, Wuhan 430064, People′s Republic of China; Tel: +86-27-59101956; E-mail: keshaoyong@163.com or shaoyong.ke@nberc.com

Supporting Information

Experimental section

Instrumentation and chemicals

All starting materials and reagents commercially available were used without further purification, unless otherwise specified. ^{1}H NMR and ^{13}C NMR spectra were recorded on a Bruker Avance III 600 MHz FT-NMR spectrometer using DMSO-d_6 or $CDCl_3$ as the solvent and tetramethylsilane (TMS) as the internal standard. Chemical shifts are reported in δ (parts per million) values, and coupling constants ^{n}J are reported in Hz. Standard abbreviations indicating multiplicity are used as follows: s = singlet, d = doublet, dd = doublet of doublets, t = triplet, q = quadruplet, m = multiplet and br = broad. Mass spectra were performed on a Waters ACQUITY UPLC® H-CLASS PDA (Waters®) instrument. Analytical thin-layer chromatography was carried out on precoated silica gel plates GF254 (Qindao Haiyang Chemical, China), and spots were visualized with ultraviolet light.

General synthetic procedure for the intermeditaes 2a-p

To a solution of dehydroepiandrosterone **1** (0.576 g, 2 mmol) in methanol (25 mL) was added appropriate aldehyde (2.1 mmol) and sodium hydroxide (0.8 g, 20 mmol), which were stirred at room temperature and detected by thin-layer chromatography. After the completion of reaction, the mixture was poured into 60 mL of ice water with stirring. Then the precipitate was filtered and dried to obtain white powder **2a-p**. Their basic physico-chemical properties and spectra data are as follows:

16-(Benzylidene)-17-oxo-5-androsten-3β-ol ***2a***

This compound was obtained following the above method as pale white powder, yield 88.6%. MS (ESI) *m/z* 377.6 $(M+H)^+$, calcd. for $C_{26}H_{32}O_2$ m/z = 376.2.

16-(2-Chlorobenzylidene)-17-oxo-5-androsten-3β-ol ***2b***

This compound was obtained following the above method as white powder, yield 90.2%. MS (ESI) *m/z* 411.5 $(M+H)^+$, calcd. for $C_{26}H_{32}ClO_2$ m/z = 410.2.

16-(4-Chlorobenzylidene)-17-oxo-5-androsten-3β-ol ***2c***

This compound was obtained following the above method as white powder, yield 97.5%. MS (ESI) *m/z* 411.5 $(M+H)^+$, calcd. for $C_{26}H_{32}ClO_2$ m/z = 410.2.

16-(2-Fluorobenzylidene)-17-oxo-5-androsten-3β-ol ***2d***

This compound was obtained following the above method as white powder, yield 93.9%. MS (ESI) *m/z* 395.6 $(M+H)^+$, calcd. for $C_{26}H_{31}FO_2$ m/z = 394.2.

16-(4-Fluorobenzylidene)-17-oxo-5-androsten-3β-ol ***2e***

This compound was obtained following the above method as white powder, yield 85.1%. MS (ESI) *m/z* 395.5 $(M+H)^+$, calcd. for $C_{26}H_{31}FO_2$ m/z = 394.2.

16-(2-Trifluoromethylbenzylidene)-17-oxo-5-androsten-3β-ol ***2f***

This compound was obtained following the above method as white powder, yield 88.9%. MS (ESI) *m/z* 445.5 $(M+H)^+$, calcd. for $C_{27}H_{31}F_3O_2$ m/z = 444.2.

16-(4-Trifluoromethylbenzylidene)-17-oxo-5-androsten-3β-ol ***2g***

This compound was obtained following the above method as white powder, yield 96.8%. MS (ESI) *m/z* 445.5 $(M+H)^+$, calcd. for $C_{27}H_{31}F_3O_2$ m/z = 444.2.

16-(2,4-Dichlorobenzylidene)-17-oxo-5-androsten-3β-ol ***2h***

This compound was obtained following the above method as white powder, yield 93.5%. MS (ESI) *m/z* 445.6 $(M+H)^+$, calcd. for $C_{26}H_{30}Cl_2O_2$ m/z = 444.2.

16-(2-Fluoro-4-bromobenzylidene)-17-oxo-5-androsten-3β-ol ***2i***

This compound was obtained following the above method as white powder, yield 83.7%. MS (ESI) *m/z* 473.5 $(M+H)^+$, calcd. for $C_{26}H_{30}BrFO_2$ m/z = 472.1.

16-(4-Methoxybenzylidene)-17-oxo-5-androsten-3β-ol ***2j***

This compound was obtained following the above method as white powder, yield 89.9%. MS (ESI) *m/z* 407.6 $(M+H)^+$, calcd. for $C_{27}H_{34}O_3$ m/z = 406.3.

16-(4-Methylthiobenzylidene)-17-oxo-5-androsten-3β-ol ***2k***

This compound was obtained following the above method as white powder, yield 93.9%. MS (ESI) *m/z* 423.5 $(M+H)^+$, calcd. for $C_{27}H_{34}O_2S$ m/z = 422.2.

16-(3,4,5-Trimethoxybenzylidene)-17-oxo-5-androsten-3β-ol ***2l***

This compound was obtained following the above method as white powder, yield 97.4%. MS (ESI) *m/z* 467.5 $(M+H)^+$, calcd. for $C_{29}H_{38}O_5$ m/z = 466.3.

16-(2-Pyridinmethylene)-17-oxo-5-androsten-3β-ol ***2m***

This compound was obtained following the above method as white powder, yield 90.3%. MS (ESI) *m/z* 378.4 $(M+H)^+$, calcd. for $C_{25}H_{31}NO_2$ m/z = 377.2.

16-(3-Pyridinmethylene)-17-oxo-5-androsten-3β-ol ***2n***

This compound was obtained following the above method as white powder, yield 98.2%. MS (ESI) *m/z* 378.3 $(M+H)^+$, calcd. for $C_{25}H_{31}NO_2$ m/z = 377.2.

16-(4-Pyridinmethylene)-17-oxo-5-androsten-3β-ol ***2o***

This compound was obtained following the above method as white powder, yield 94.2%. MS (ESI) *m/z* 378.3 $(M+H)^+$, calcd. for $C_{25}H_{31}NO_2$ m/z = 377.2.

16-(3-Phenoxybenzylidene)-17-oxo-5-androsten-3β-ol ***2p***

This compound was obtained following the above method as white powder, yield 92.7%. MS (ESI) *m/z* 469.5 $(M+H)^+$, calcd. for $C_{32}H_{36}O_3$ m/z = 468.3.

General synthetic procedure for target compounds 3a-p

To a solution of substituted benzylidene-dehydroepiandrosterone derivatives **2a-p** (1 mmol) in t-butanol (15 mL) was added guanidine nitrate (0.488 g, 4 mmol) and potassium *t*-butoxide (0.336 g, 3 mmol), which were heated under reflux overnight. After the completion of reaction, the mixtures was poured into 50 mL of ice water with stirring, and tune the pH value to 7-8, and then the precipitate was filtered and washed with water, dried under reduced pressure to obtain the target compounds **3a-p**. All the compounds were characterized by ESI-MS, ^{1}H NMR and ^{13}C NMR spectroscopic data. Their physico-chemical properties and spectra data are as follows:

3β-Hydroxy-2'-amino-4'-(phenyl)-androst-[16,17-e]pyrimidine ***3a***

This compound was obtained following the above method as yellowish solid, yield 76.4%. ^{1}H NMR (600 MHz, DMSO-d_6): δ = 7.82 (d, J = 6 Hz, 2H), 7.50-7.45 (m, 3H), 6.51 (s, 2H), 5.30 (s, 1H), 4.67 (d, J = 3.6 Hz, 1H), 3.26 (bs, 1H), 2.76-2.55 (m, 2H), 2.18-1.97 (m, 4H), 1.80-1.58 (m, 7H), 1.49-1.33 (m, 4H), 1.02 (s, 3H), 0.99 (s, 3H); ^{13}C NMR (150 MHz, DMSO-d_6): δ = 163.45, 159.50, 141.92, 138.49, 129.82, 128.73, 128.63, 120.71, 118.41, 70.44, 55.46, 51.32, 50.54, 45.65, 42.72, 37.27, 36.77, 33.39, 31.93, 31.87, 31.04, 30.68, 29.89, 20.67, 19.65, 17.30, 13.64; MS (ESI) *m/z* 416.6 $(M+H)^+$, calcd. for $C_{27}H_{33}N_3O$ m/z = 415.3.

3β-Hydroxy-2'-amino-4'-(2-chlorophenyl)-androst-[16,17-e]pyrimidine ***3b***

This compound was obtained following the above method as white powder, yield 95.7%. ^{1}H NMR (600 MHz, DMSO-d_6): δ = 7.55-7.40 (m, 3H), 7.05 (bs, 1H), 6.59 (s, 1H), 5.26 (d, J = 6 Hz, 1H), 4.66 (d, J = 6 Hz, 1H), 3.27-3.23 (m, 1H), 2.39-2.31 (m, 1H), 2.25-1.90 (m, 5H), 1.80-1.30 (m, 9H), 1.10-0.90 (m, 7H); MS (ESI) *m/z* 450.3 $(M+H)^+$, calcd. for $C_{27}H_{32}ClN_3O$ m/z = 449.2.

3β-Hydroxy-2'-amino-4'-(4-chlorophenyl)-androst-[16,17-e]pyrimidine ***3c***

This compound was obtained following the above method as white powder, yield 80.2%. ^{1}H NMR (600 MHz, $CDCl_3$): δ = 7.77 (d, J = 8.4 Hz, 2H), 7.45 (d, J = 9 Hz, 2H), 5.38 (d, J = 4.8 Hz, 1H), 5.19 (s, 1H), 3.54 (bs, 1H),

2.75-2.62 (m, 2H), 2.34-2.25 (m, 2H), 2.18-2.11 (m, 2H), 1.90-1.50 (m, 12H), 1.12-1.08 (m, 6H); ^{13}C NMR (150 MHz, $CDCl_3$): δ = 162.00, 141.16, 135.83, 129.75, 128.71, 120.95, 120.22, 71.60, 55.66, 50.48, 45.73, 42.20, 37.09, 36.73, 32.89, 31.57, 31.25, 30.67, 29.90, 20.49, 19.44, 16.86; MS (ESI) *m/z* 450.5 $(M+H)^+$, calcd. for $C_{27}H_{32}ClN_3O$ m/z = 449.2.

3β-Hydroxy-2'-amino-4'-(2-fluorophenyl)-androst-[16,17-e]pyrimidine ***3d***

This compound was obtained following the above method as white solid, yield 77.6%. ^{1}H NMR (600 MHz, DMSO-d_6): δ = 7.56-7.29 (m, 3H), 7.06 (bs, 1H), 6.60 (s, 1H), 5.27 (s, 1H), 4.66 (d, J = 3.6 Hz, 1H), 3.28-3.22 (m, 1H), 2.45-2.25 (m, 2H), 2.20-1.92 (m, 4H), 1.82-1.30 (m, 10H), 1.08-0.90 (m, 7H); MS (ESI) *m/z* 434.3 $(M+H)^+$, calcd. for $C_{27}H_{32}FN_3O$ m/z = 433.3.

3β-Hydroxy-2'-amino-4'-(4-fluorophenyl)-androst-[16,17-e]pyrimidine ***3e***

This compound was obtained following the above method as white powder, yield 72.3%. ^{1}H NMR (600 MHz, DMSO-d_6): δ = 7.89-7.87 (m, 2H), 7.32 (t, J = 8.4 Hz, 2H), 6.53 (s, 1H), 5.30 (d, J = 3.6 Hz, 1H), 4.67 (d, J = 4.8 Hz, 1H), 3.28-3.24 (m, 1H), 2.75-2.58 (m, 2H), 2.19-2.02 (m, 4H), 1.80-1.34 (m, 9H), 1.06-0.92 (m, 7H); MS (ESI) *m/z* 434.4 $(M+H)^+$, calcd. for $C_{27}H_{32}FN_3O$ m/z = 433.3.

3β-Hydroxy-2'-amino-4'-(2-trifluoromethylphenyl)-androst-[16,17-e]pyrimidine ***3f***

This compound was obtained following the above method as white powder, yield 70.1%. ^{1}H NMR (600 MHz, DMSO-d_6): δ = 7.84-7.42 (m, 4H), 6.56 (s, 1H), 5.24 (bs, 1H), 4.65 (d, J = 3.6 Hz, 1H), 3.29-3.20 (m, 1H), 2.17-2.03 (m, 4H), 1.93-1.18 (m, 12H), 1.05-0.90 (m, 7H); MS (ESI) *m/z* 484.4 $(M+H)^+$, calcd. for $C_{28}H_{32}F_3N_3O$ m/z = 483.2.

3β-Hydroxy-2'-amino-4'-(4-trifluoromethyl phenyl)-androst-[16,17-e]pyrimidine ***3g***

This compound was obtained following the above method as white powder, yield 77.6%. ^{1}H NMR (600 MHz, $CDCl_3$): δ = 7.92 (d, J = 8.4 Hz, 2H), 7.73 (d, J = 8.4 Hz, 2H), 5.38 (d, J = 4.8 Hz, 1H), 5.13 (s, 2H), 3.57-3.51 (m, 1H), 2.75-2.65 (m, 2H), 2.34-2.23 (m, 2H), 2.20-2.07 (m, 2H), 1.90-1.45 (m, 12H), 1.10-1.09 (m, 6H); ^{13}C NMR (150 MHz, $CDCl_3$): δ = 162.37, 158.79, 141.41, 141.19, 128.73, 125.38, 120.97, 120.72, 71.63, 55.72, 50.52, 45.76, 42.23, 37.13, 36.77, 32.93, 31.59, 31.29, 30.71, 29.79, 20.53, 19.47, 16.92; MS (ESI) *m/z* 484.4 $(M+H)^+$, calcd. for $C_{28}H_{32}F_3N_3O$ m/z = 483.2.

3β-Hydroxy-2'-amino-4'-(2,4-dichlorophenyl)-androst-[16,17-e]pyrimidine ***3h***

This compound was obtained following the above method as white solid, yield 88.2%. ^{1}H NMR (600 MHz, DMSO-d_6): δ = 7.74 (s, 1H), 7.52 (d, J = 8.4 Hz, 1H), 7.45 (d, J = 8.4 Hz, 1H), 6.65 (s, 1H), 5.26 (bs, 1H), 4.66 (s, 1H), 3.25 (bs, 1H), 2.36-1.90 (m, 5H), 1.80-1.30 (m, 10H), 1.10-0.85 (m, 8H); ^{13}C NMR (150 MHz, DMSO-d_6): δ = 163.35, 158.97, 141.92, 136.74, 134.40, 132.58, 129.46, 127.91, 120.64, 70.41, 55.01, 50.46, 45.97, 42.68, 37.25, 36.78, 33.18, 31.04, 30.65, 28.13, 20.63, 19.60, 17.31; MS (ESI) *m/z* 484.4 $(M+H)^+$, calcd. for $C_{27}H_{31}Cl_2N_3O$ m/z = 483.2.

3β-Hydroxy-2'-amino-4'-(2-fluoro-4-bromophenyl)-androst-[16,17-e]pyrimidine ***3i***

This compound was obtained following the above method as white solid, yield 81.4%. ^{1}H NMR (600 MHz, DMSO-d_6): δ = 7.69 (d, J = 9.6 Hz, 1H), 7.55-7.50 (m, 2H), 6.65 (s, 1H), 5.27 (s, 1H), 4.66 (d, J = 4.2 Hz, 1H), 3.28-3.22 (m, 1H), 2.43-2.27 (m, 2H), 2.20-1.95 (m, 4H), 1.80-1.33 (m, 10H), 1.05-0.92 (m, 8H); ^{13}C NMR (150 MHz, DMSO-d_6): δ = 163.43, 160.22, 158.58, 155.65, 141.87, 132.96, 128.13, 125.75, 123.19, 120.67, 120.57,

119.90, 70.41, 54.99, 50.45, 45.95, 42.69, 37.24, 36.77, 33.25, 31.91, 31.01, 30.64, 28.50, 20.62, 19.61, 17.27; MS (ESI) *m/z* 512.4 $(M+H)^+$, calcd. for $C_{27}H_{31}BrFN_3O$ m/z = 511.2.

3β-Hydroxy-2'-amino-4'-(4-methoxyphenyl)-androst-[16,17-e]pyrimidine ***3j***

This compound was obtained following the above method as white powder, yield 80.9%. ^1H NMR (600 MHz, DMSO-d_6): δ = 7.80 (d, J = 8.4 Hz, 2H), 7.04 (d, J = 9.0 Hz, 2H), 6.44 (s, 2H), 5.31 (s, 1H), 4.67 (d, J = 3.6 Hz, 1H), 3.81 (s, 3H), 3.25 (bs, 1H), 2.72-2.60 (m, 2H), 2.20-2.00 (m, 4H), 1.80-1.32 (m, 10H), 1.02-0.95 (m, 7H); ^{13}C NMR (150 MHz, DMSO-d_6): δ = 163.33, 160.56, 158.95, 141.94, 130.72, 130.08, 120.65, 117.64, 114.10, 70.45, 55.65, 55.41, 50.53, 45.53, 42.73, 37.22, 36.71, 33.42, 31.94, 31.05, 30.68, 30.08, 20.62, 19.65, 17.21; MS (ESI) *m/z* 446.5 $(M+H)^+$, calcd. for $C_{28}H_{35}N_3O_2$ m/z = 445.3.

3β-Hydroxy-2'-amino-4'-(4-methylthio phenyl)-androst-[16,17-e]pyrimidine ***3k***

This compound was obtained following the above method as white powder, yield 71.6%. ^1H NMR (600 MHz, DMSO-d_6): δ = 7.78 (d, J = 8.4 Hz, 2H), 7.35 (d, J = 8.4 Hz, 2H), 6.49 (s, 2H), 5.30 (s, 1H), 4.67 (d, J = 4.2 Hz, 1H), 3.27-3.24 (m, 1H), 2.73-2.58 (m, 2H), 2.52 (s, 3H), 2.18-2.01 (m, 4H), 1.80-1.56 (m, 6H), 1.48-1.34 (m, 4H), 1.02-0.96 (m, 7H); ^{13}C NMR (150 MHz, DMSO-d_6): δ = 163.38, 158.86, 141.93, 140.62, 134.74, 129.08, 125.58, 120.71, 118.08, 70.44, 55.44, 50.54, 45.60, 42.71, 37.26, 36.77, 33.39, 31.92, 31.04, 30.67, 30.00, 20.67, 19.64, 17.29, 14.70; MS (ESI) *m/z* 462.3 $(M+H)^+$, calcd. for $C_{28}H_{35}N_3OS$ m/z = 461.3.

3β-Hydroxy-2'-amino-4'-(3,4,5-trimethoxy phenyl)-androst-[16,17-e]pyrimidine ***3l***

This compound was obtained following the above method as white powder, yield 72.7%. ^1H NMR (600 MHz, DMSO-d_6): δ = 7.07 (s, 2H), 6.49 (s, 2H), 5.30 (s, 1H), 4.67 (d, J = 4.2 Hz, 1H), 3.83 (s, 6H), 3.71 (s, 3H), 3.28-3.22 (m, 1H), 2.79-2.61 (m, 2H), 2.18-2.02 (m, 4H), 1.80-1.57 (m, 6H), 1.46-1.33 (m, 3H), 1.02-0.97 (m, 8H); ^{13}C NMR (150 MHz, DMSO-d_6): δ = 163.33, 159.40, 153.07, 141.99, 138.87, 134.00, 120.65, 118.36, 106.02, 70.45, 60.57, 56.36, 55.52, 50.53, 45.70, 42.75, 37.25, 36.79, 33.40, 31.92, 30.99, 30.67, 29.98, 20.66, 19.68, 17.28; MS (ESI) *m/z* 506.4 $(M+H)^+$, calcd. for $C_{30}H_{39}N_3O_4$ m/z = 505.3.

3β-Hydroxy-2'-amino-4'-(2-pyridinyl)-androst-[16,17-e]pyrimidine ***3m***

This compound was obtained following the above method as white powder, yield 76.9%. ^1H NMR (600 MHz, DMSO-d_6): δ = 8.70 (d, J = 4.2 Hz, 1H), 8.21 (d, J = 7.8 Hz, 1H), 7.95-7.92 (m, 1H), 7.46-7.44 (m, 1H), 6.56 (s, 2H), 5.32 (s, 1H), 4.67 (d, J = 4.2 Hz, 1H), 3.29-3.23 (m, 1H), 3.19-3.15 (m, 1H), 2.64-2.59 (m, 1H), 2.19-2.04 (m, 4H), 1.80-1.34 (m, 9H), 1.07-0.90 (m, 8H); ^{13}C NMR (150 MHz, DMSO-d_6): δ = 163.23, 157.35, 156.83, 149.39, 141.88, 137.27, 124.60, 122.80, 120.83, 119.52, 70.44, 55.08, 50.60, 45.39, 42.72, 37.29, 36.78, 33.41, 31.93, 31.77, 31.22, 30.71, 30.46, 20.71, 19.63, 17.34; MS (ESI) *m/z* 417.4 $(M+H)^+$, calcd. for $C_{26}H_{32}N_4O$ m/z = 416.3.

3β-Hydroxy-2'-amino-4'-(3-pyridinyl)-androst-[16,17-e]pyrimidine ***3n***

This compound was obtained following the above method as white powder, yield 68.1%. ^1H NMR (600 MHz, DMSO-d_6): δ = 8.99 (d, J = 2.4 Hz, 1H), 8.65 (q, J = 4.8 Hz, 1H), 8.18-8.16 (m, 1H), 7.53 (q, J = 4.8 Hz, 1H), 6.62 (s, 2H), 5.30 (d, J = 4.8 Hz, 1H), 4.67 (d, J = 4.2 Hz, 1H), 3.28-3.24 (m, 1H), 2.77-2.60 (m, 2H), 2.19-2.03 (m, 4H), 1.80-1.34 (m, 10H), 1.06-0.90 (m, 7H); ^{13}C NMR (150 MHz, DMSO-d_6): δ = 163.52, 157.10, 150.57, 149.46, 141.93, 135.95, 134.02, 123.97, 120.69, 118.92, 70.43, 55.40, 50.52, 45.72, 42.71, 37.20, 36.78, 33.31, 31.92, 31.01, 30.69, 29.50, 20.65, 19.65, 17.30; MS (ESI) *m/z* 417.4 $(M+H)^+$, calcd. for $C_{26}H_{32}N_4O$ m/z = 416.3.

3β-Hydroxy-2'-amino-4'-(4-pyridinyl)-androst-[16,17-e]pyrimidine ***3o***

This compound was obtained following the above method as white powder, yield 65.1%. ^{1}H NMR (600 MHz, DMSO-d_6): δ = 8.71 (d, J = 4.8 Hz, 2H), 7.76 (d, J = 4.8 Hz, 2H), 6.68 (s, 2H), 5.30 (s, 1H), 4.68 (s, 1H), 3.26 (bs, 1H), 2.76-2.64 (m, 2H), 2.19-2.03 (m, 4H), 1.80-1.34 (m, 10H), 1.05-0.90 (m, 7H); ^{13}C NMR (150 MHz, DMSO-d_6): δ = 163.57, 156.99, 150.43, 150.40, 145.59, 141.94, 122.84, 120.68, 119.33, 70.43, 55.29, 50.50, 45.70, 42.71, 37.26, 36.78, 33.29, 31.92, 31.04, 30.70, 29.49, 20.65, 19.65, 17.31; MS (ESI) *m/z* 417.4 $(M+H)^+$, calcd. for $C_{26}H_{32}N_4O$ m/z = 416.3.

3β-Hydroxy-2'-amino-4'-(3-phenoxy)-androst-[16,17-e]pyrimidine ***3p***

This compound was obtained following the above method as white powder, yield 73.6%. ^{1}H NMR (600 MHz, DMSO-d_6): δ = 7.59 (d, J = 7.8 Hz, 1H), 7.51 (t, J = 7.8 Hz, 1H), 7.43-7.40 (m, 3H), 7.19-7.13 (m, 2H), 7.07 (d, J = 7.8 Hz, 2H), 6.50 (s, 2H), 5.32 (s, 1H), 4.72 (s, 1H), 3.26 (bs, 1H), 2.60-2.56 (m, 2H), 2.19-2.00 (m, 4H), 1.79-1.34 (m, 10H), 1.02-0.90 (m, 7H); ^{13}C NMR (150 MHz, DMSO-d_6): δ = 163.36, 158.53, 157.25, 156.92, 141.90, 140.35, 130.62, 130.50, 124.18, 123.72, 120.72, 120.11, 119.32, 118.45, 118.26, 70.44, 55.33, 50.49, 45.63, 42.67, 37.25, 36.76, 33.33, 31.88, 31.03, 30.65, 29.88, 20.63, 19.63, 17.26; MS (ESI) *m/z* 508.7 $(M+H)^+$, calcd. for $C_{33}H_{37}N_3O_2$ m/z = 507.3.

In vitro cytotoxicity assay

The in vitro cytotoxicity of the synthesized compounds **2a-p** and **3a-p** against various human cancer cell lines was measured by the MTT [3-(4,5-dimethylthiazol-2-yl)-2,5-diphenyltetrazoliumbromide] colorimetric method[35], and the general procedures were previously reported in literatures[36-38]. All the data of the experiment were analyzed according to SPSS software, and the 50% inhibitory concentrations (IC_{50}) of each compound for the different cell lines were determined. All assays were performed in triplicate on three independent experiments, and measurement data were expressed as the mean ± S. D.

Spectroscopy for target compounds

Structures of target compounds **3a-p** were confirmed by their ^{1}H NMR, ^{13}C NMR, ESI-MS, and their ^{1}H NMR, ^{13}C NMR, and ESI-MS were consistent with the assigned structures. The typical ^{1}H NMR and ^{13}C NMR spectra for synthesized compounds have been presented in the following, which can confirm the result.

Compd 3a

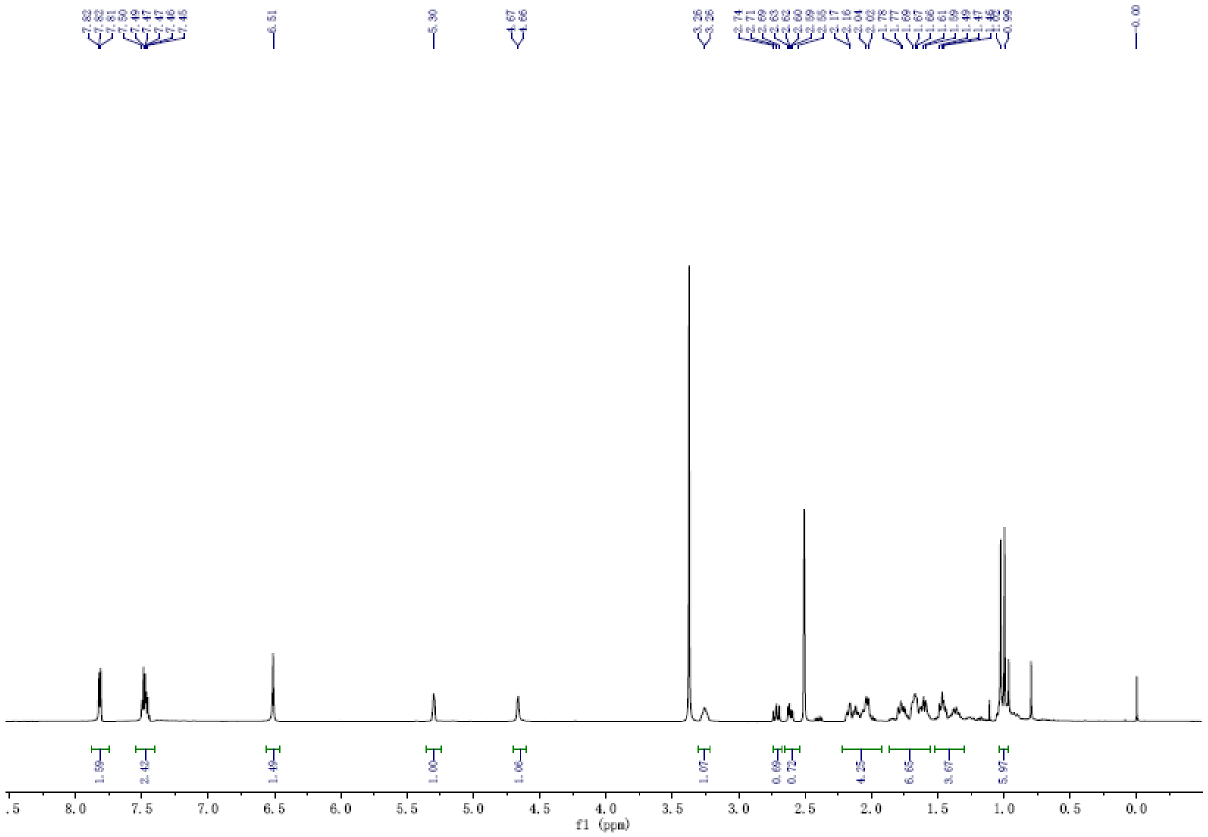

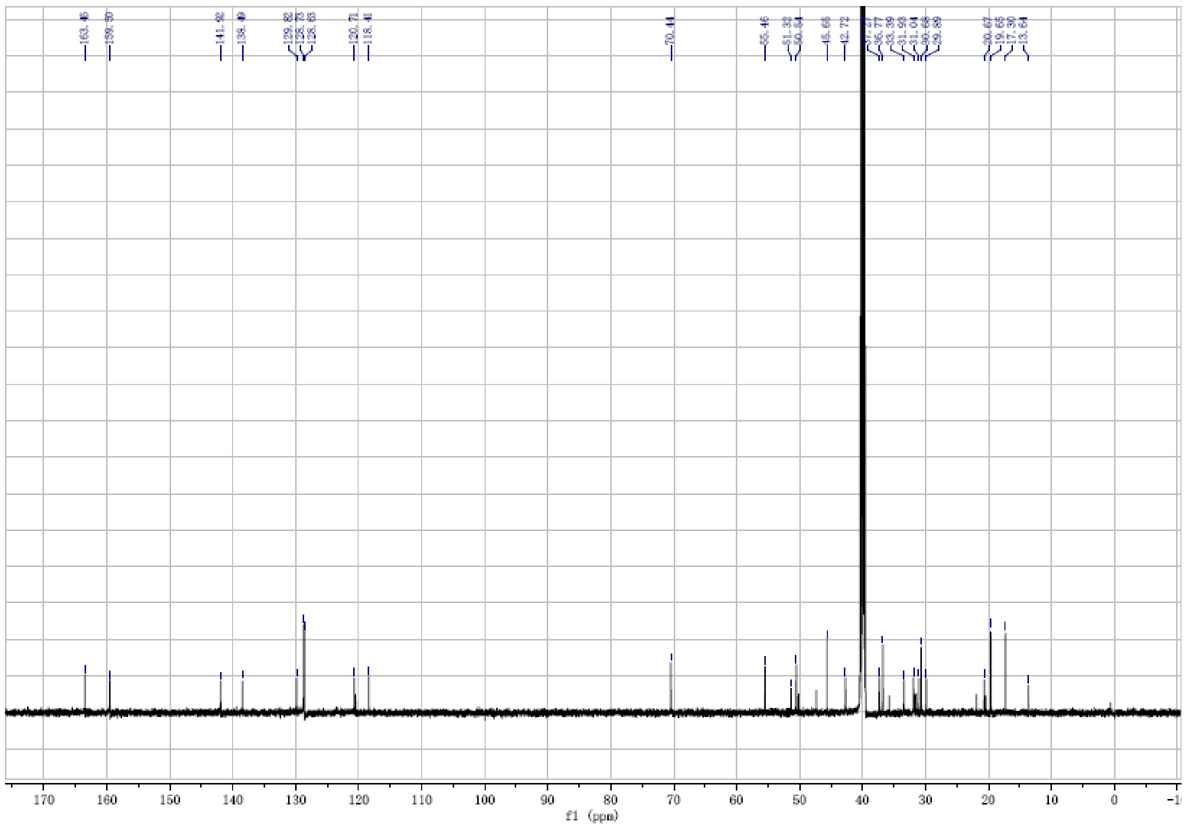

Compd 3b

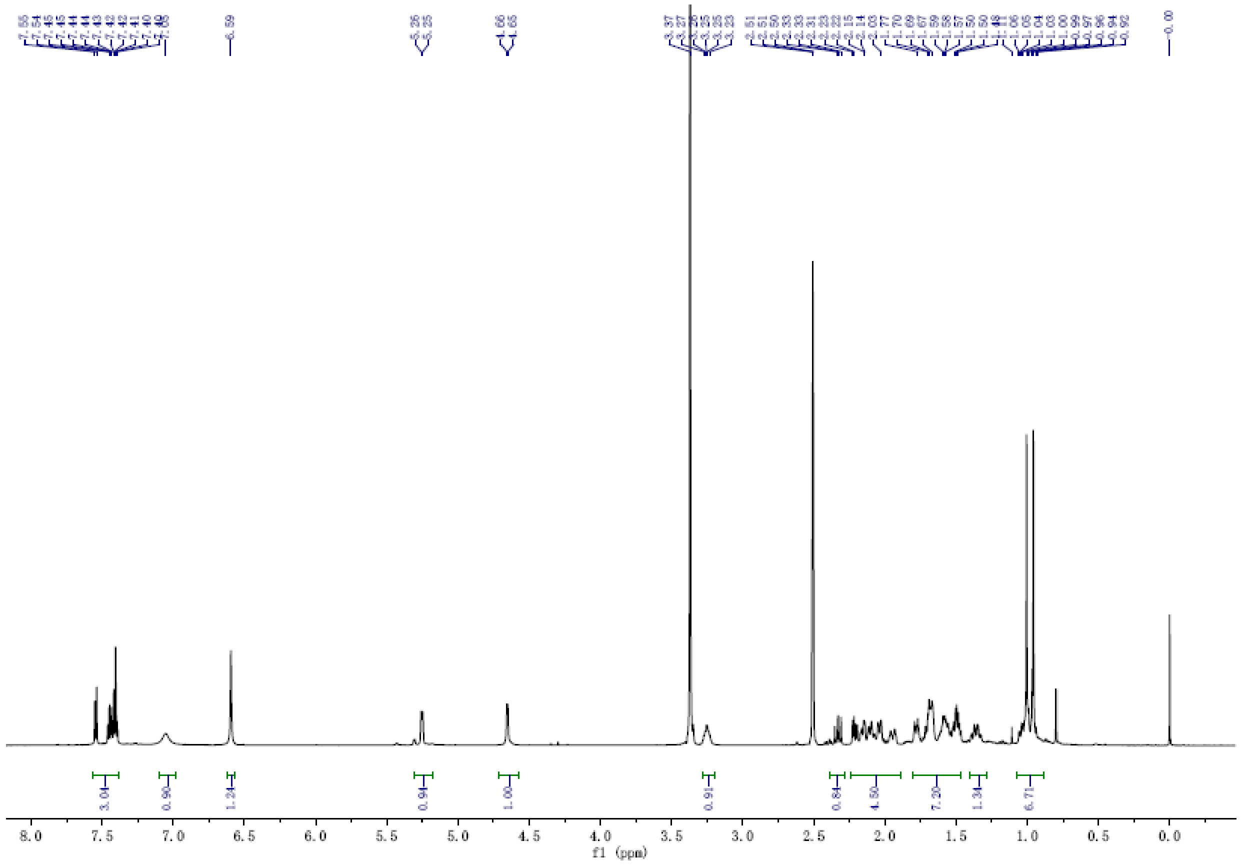

Compd 3c

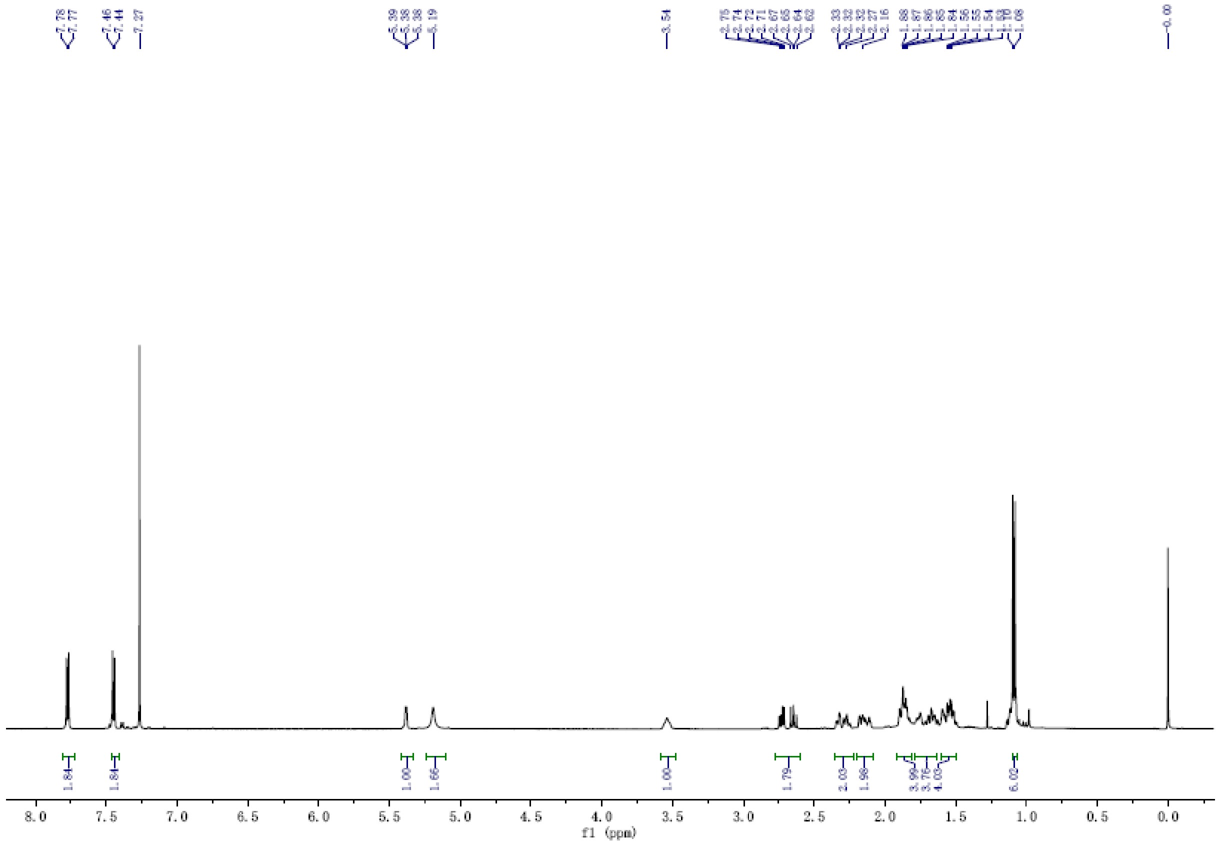

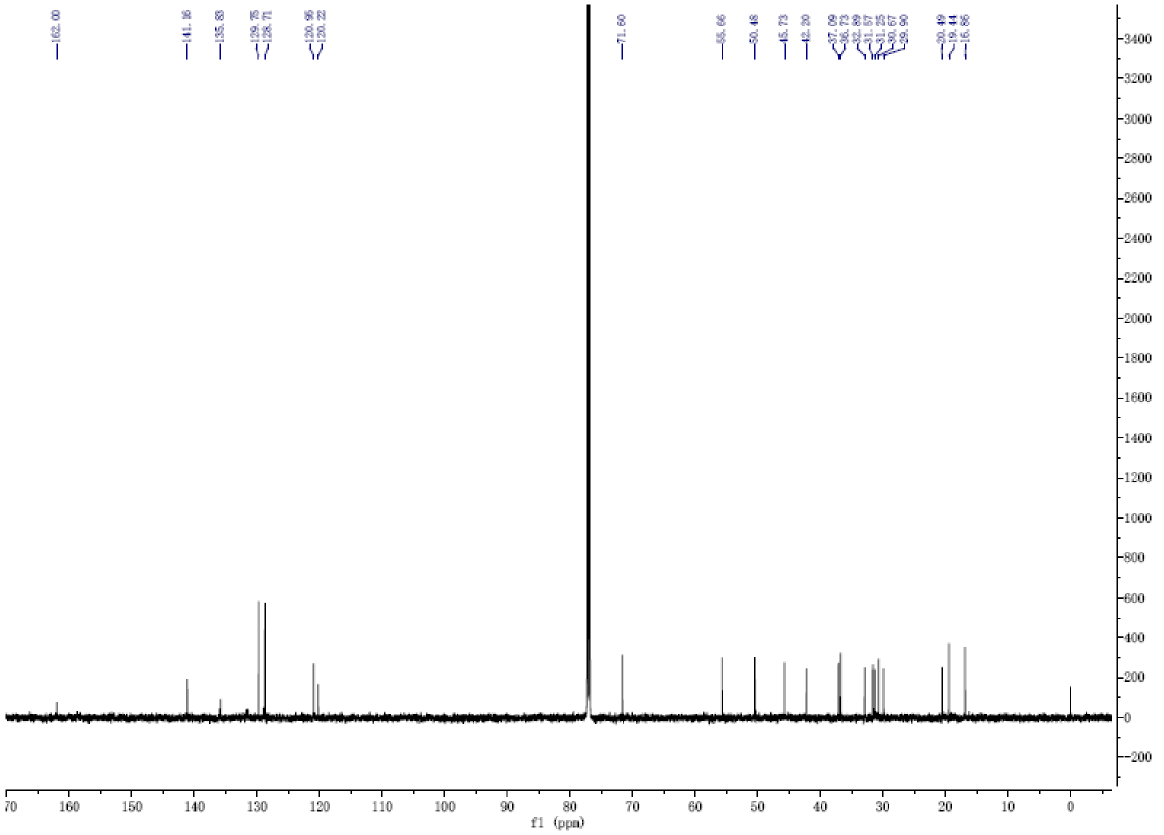

Compd 3d

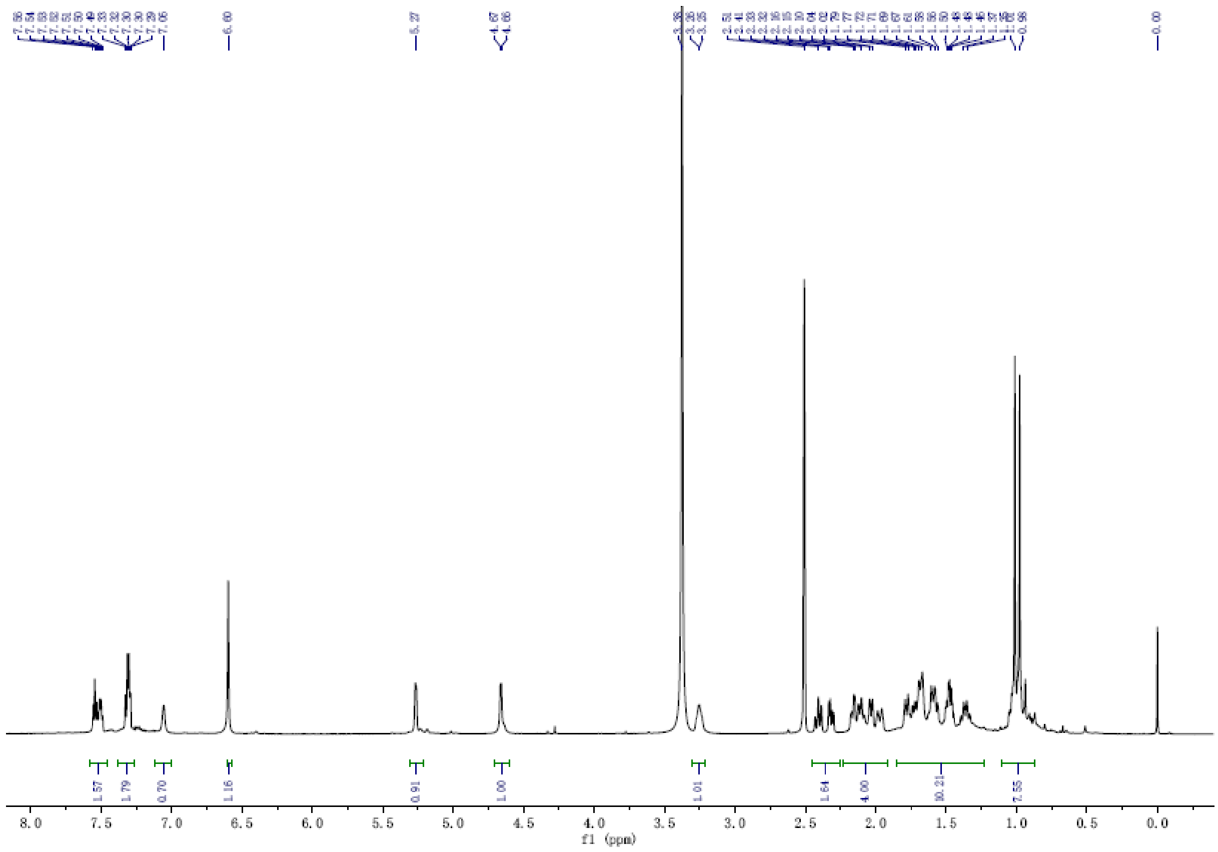

Compd 3e

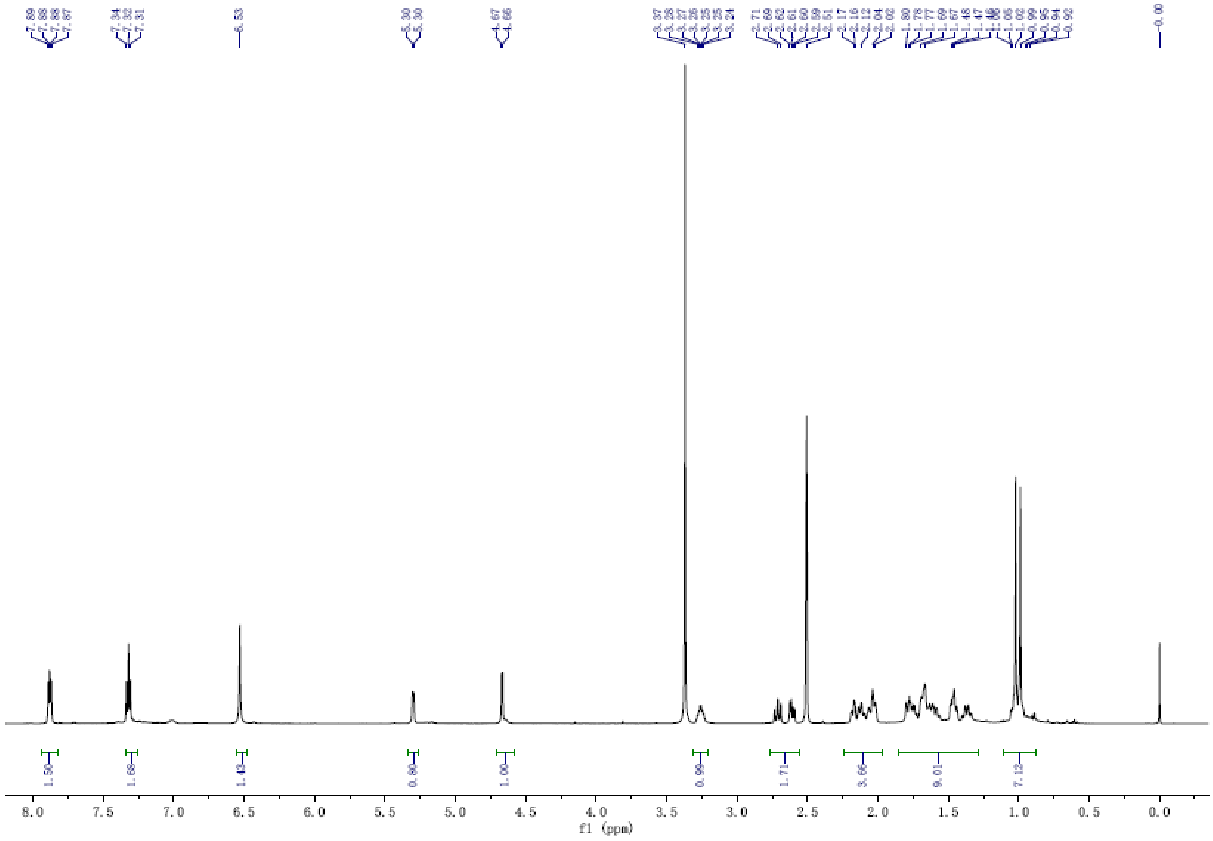
7.89
7.88
7.88
7.87
7.34
7.32
7.31
6.53
5.30
5.30
4.67
4.66
0.00
1.50
1.68
1.43
0.80
1.00
0.99
1.71
3.66
9.01
7.12
8.0
7.5
7.0
6.5
6.0
5.5
5.0
4.5
4.0
3.5
3.0
2.5
2.0
1.5
1.0
0.5
0.0
f1 (ppm)

Compd 3f

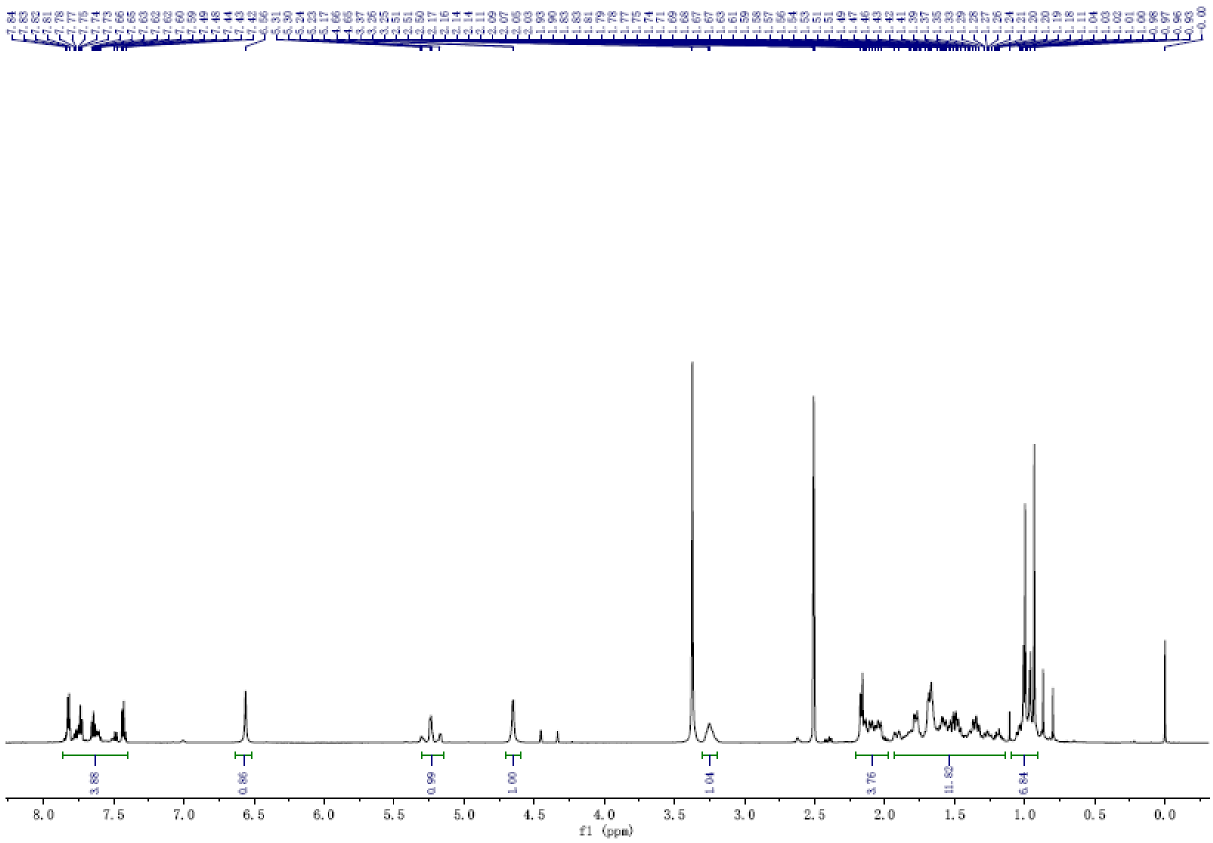

Compd 3g

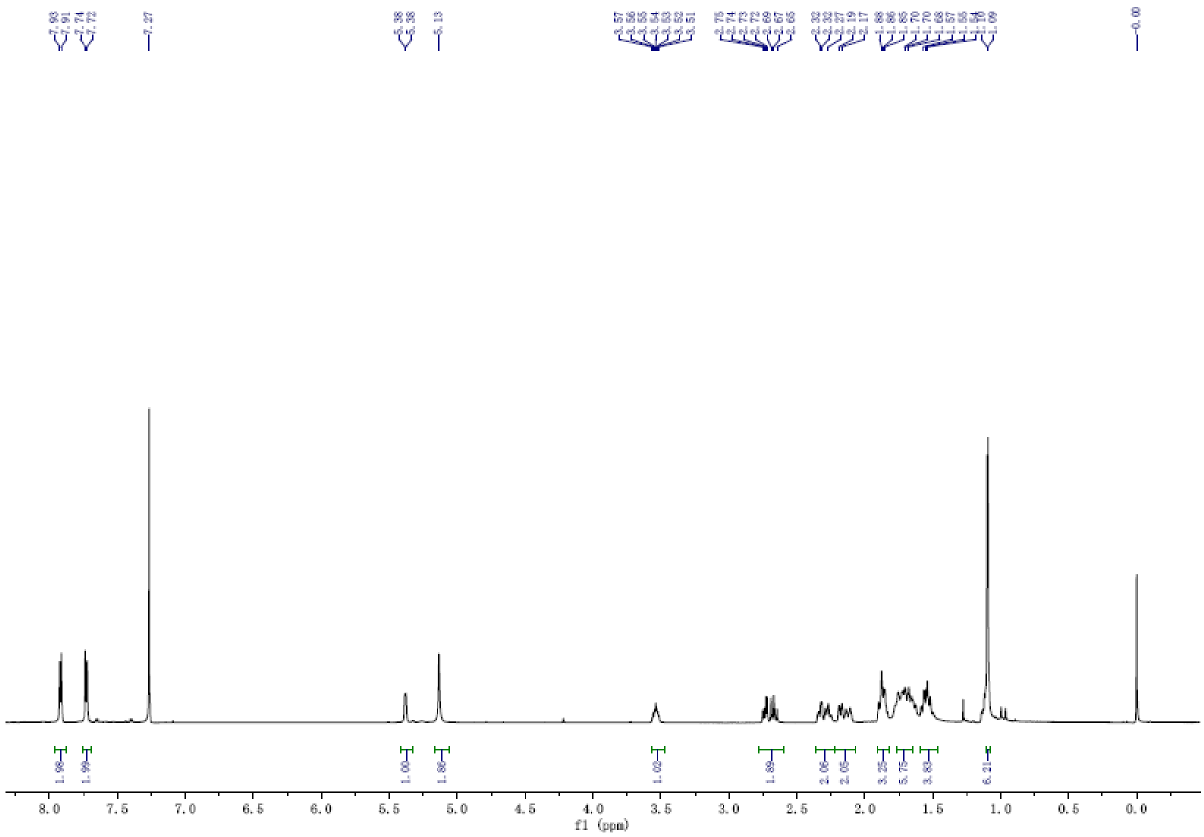

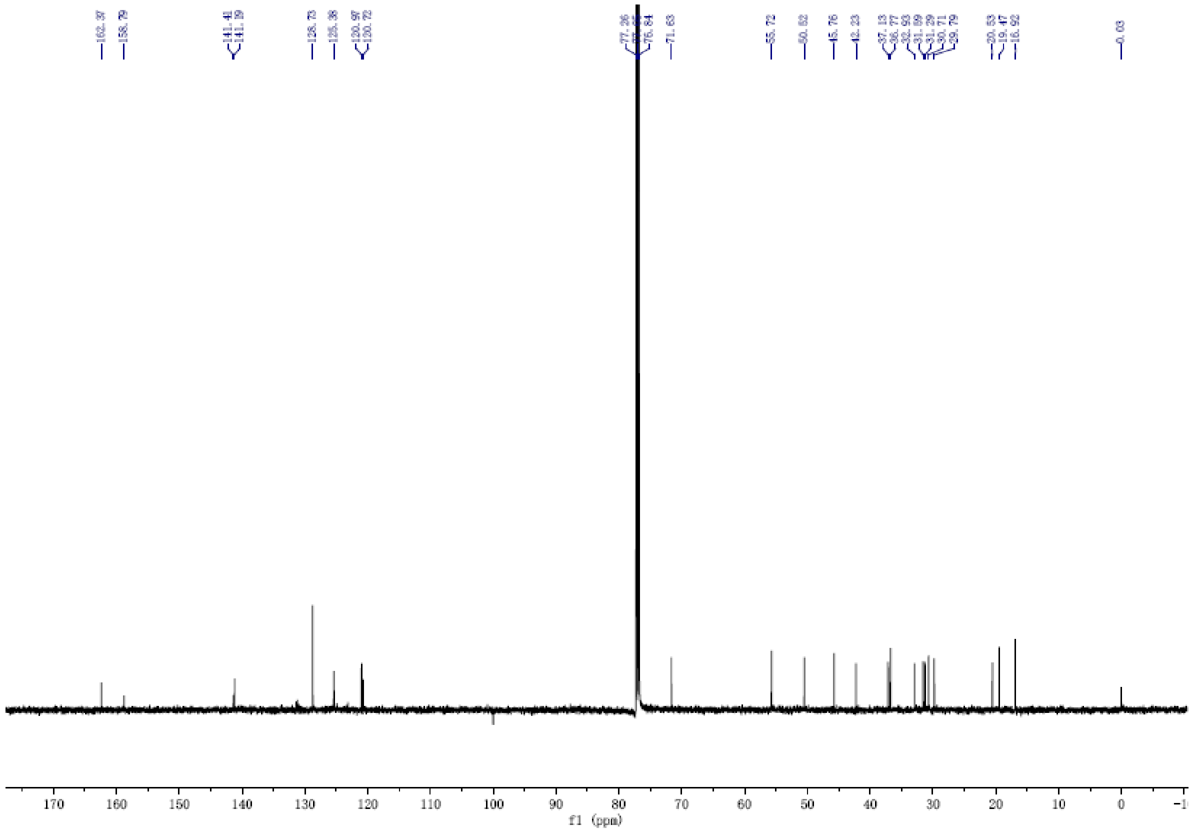

Compd 3h

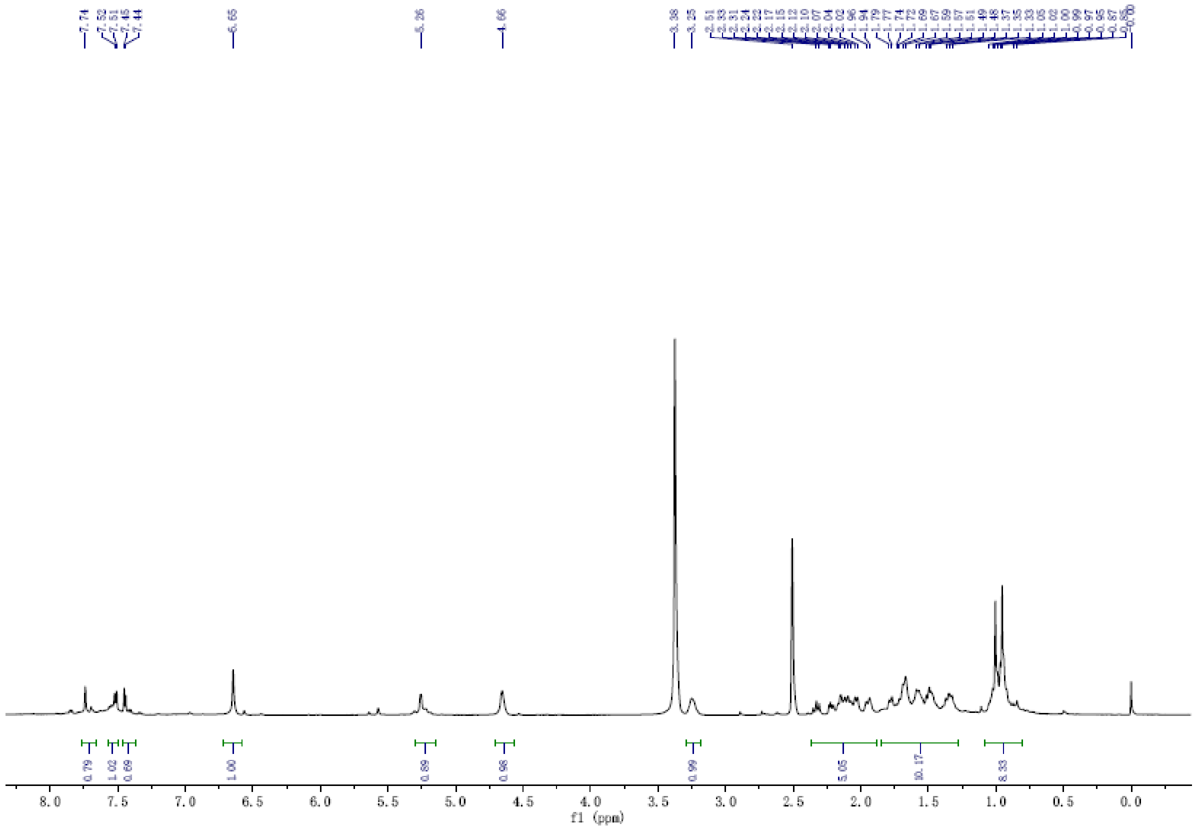

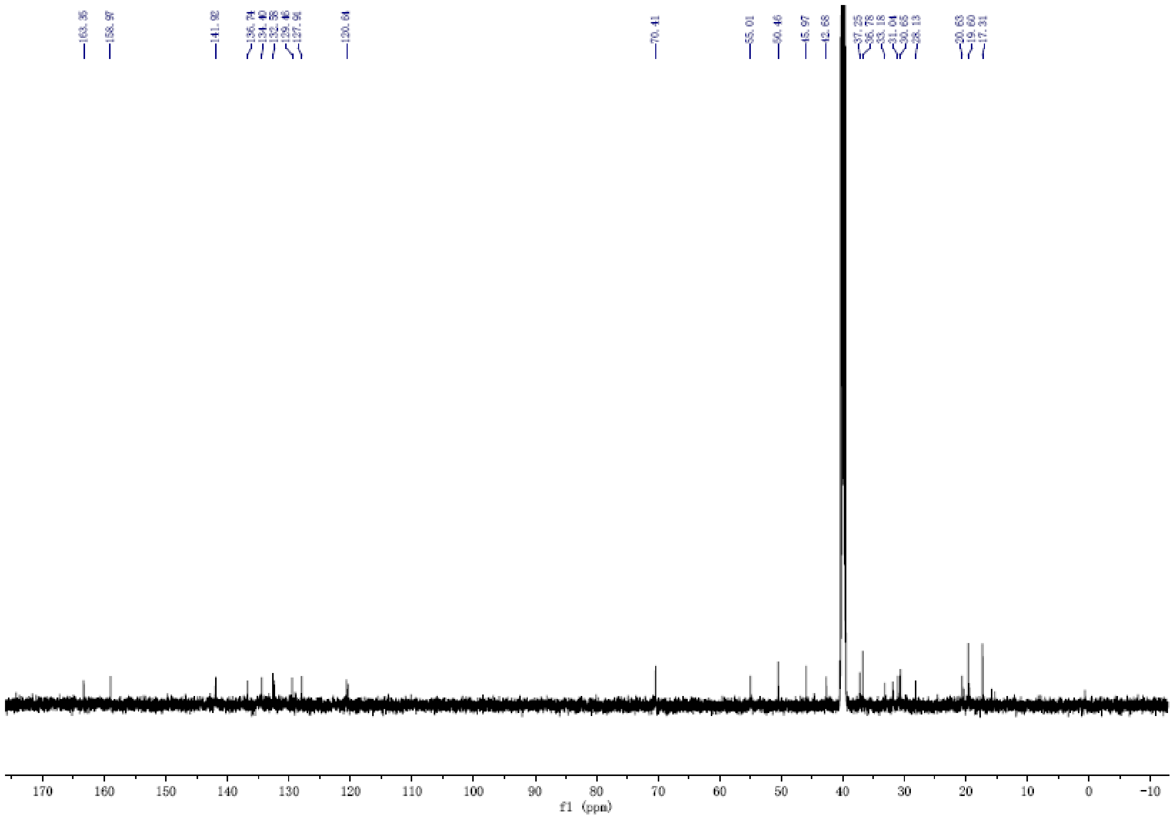

Compd 3i

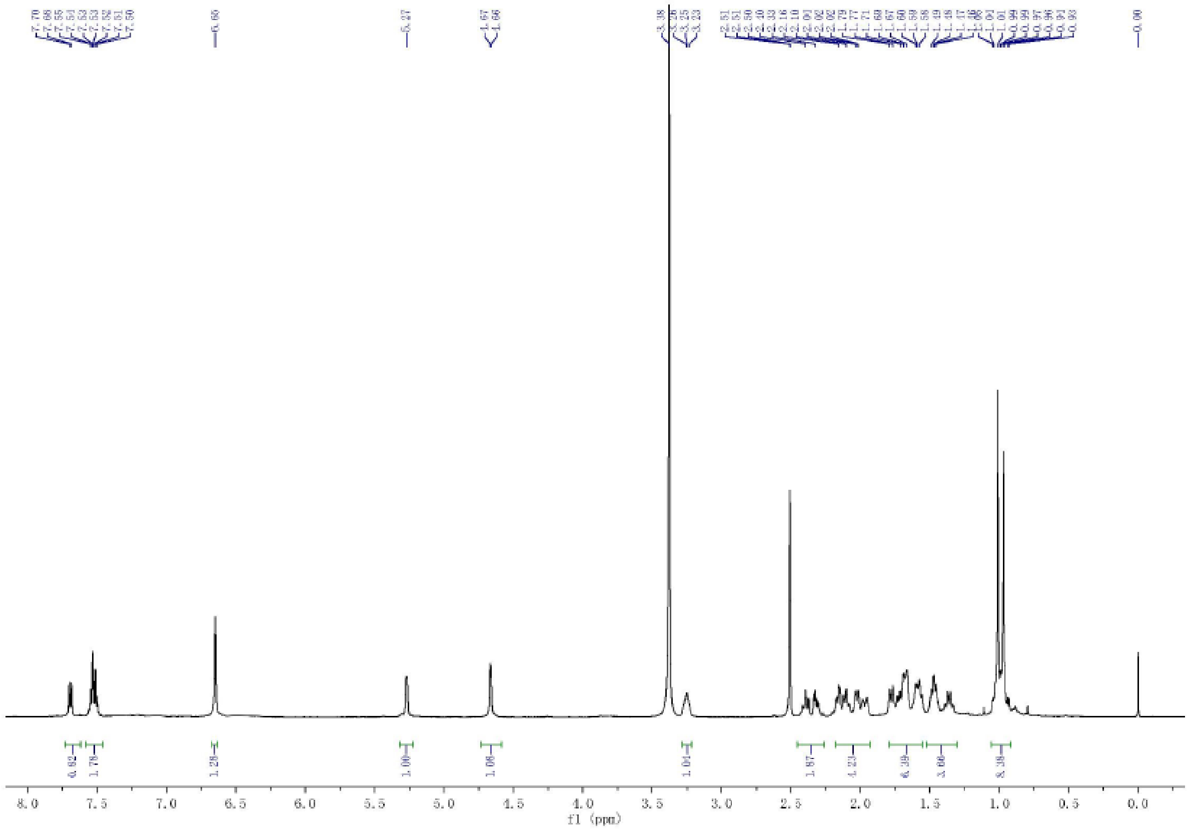

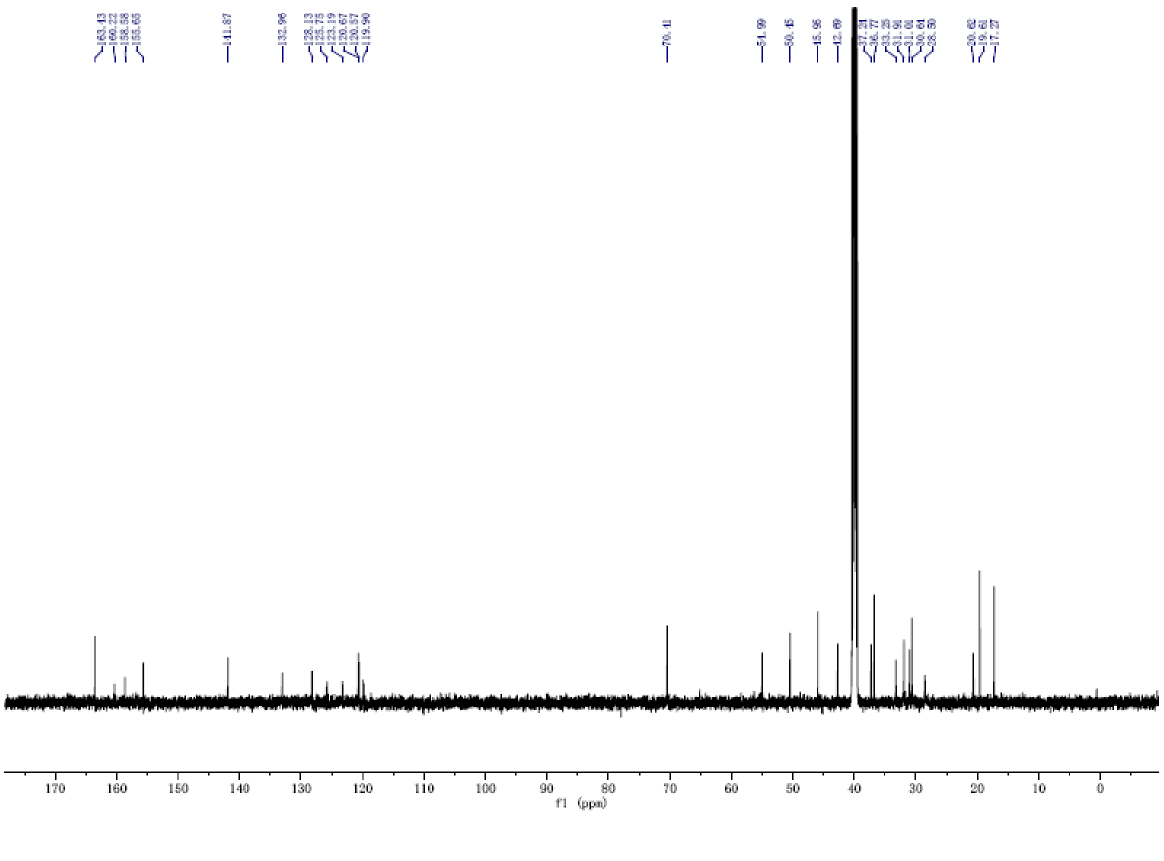

Compd 3j

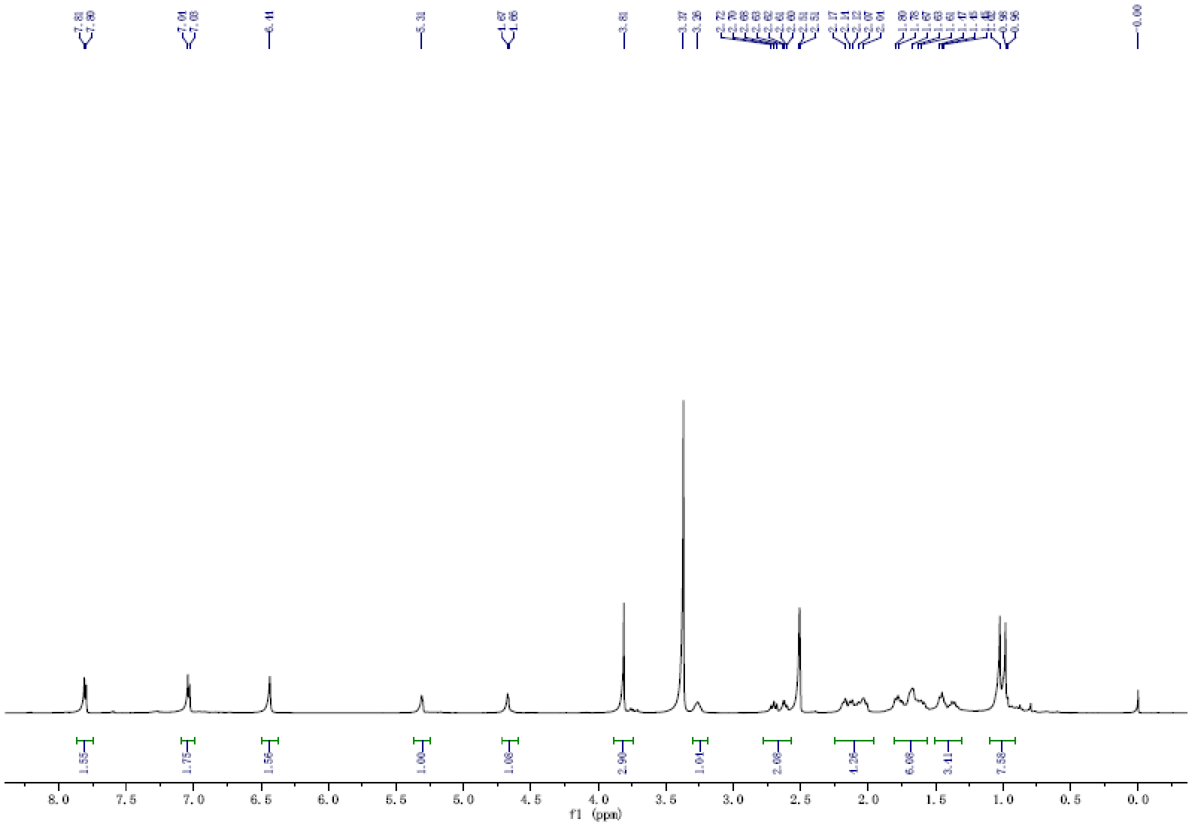

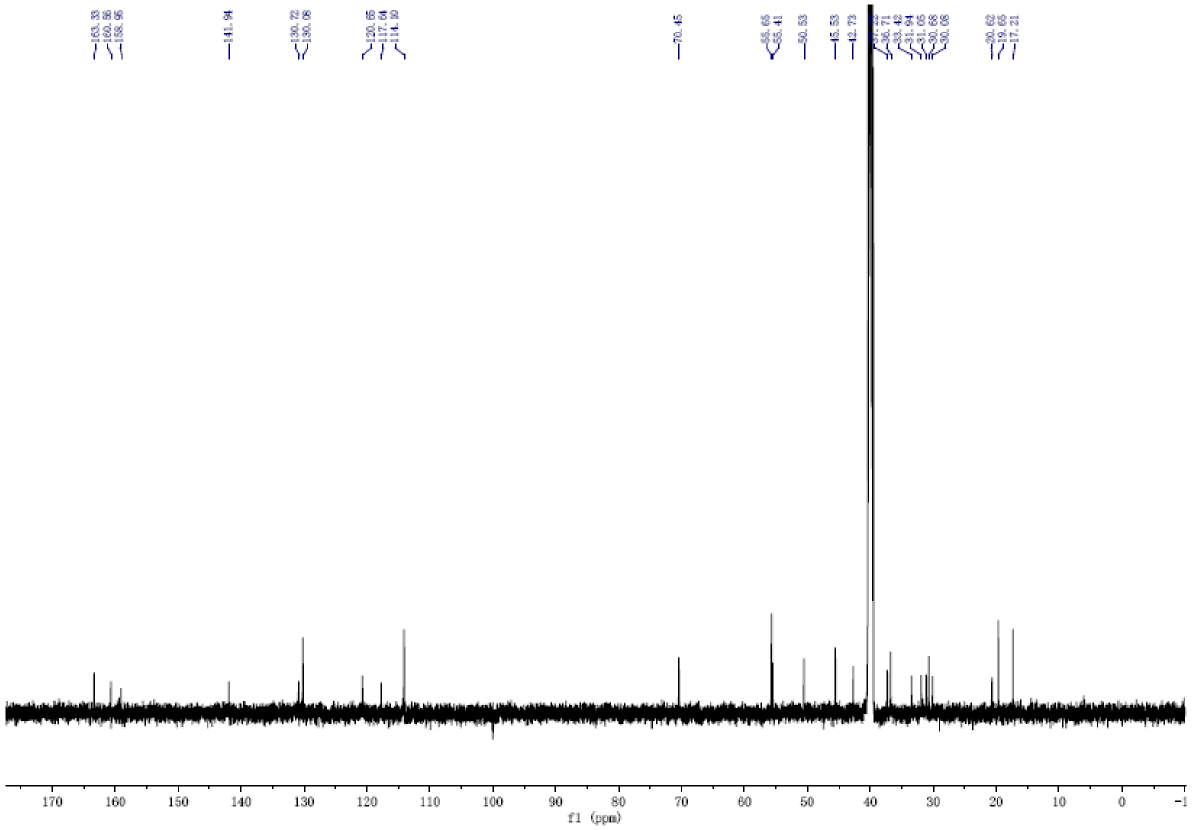

Compd 3k

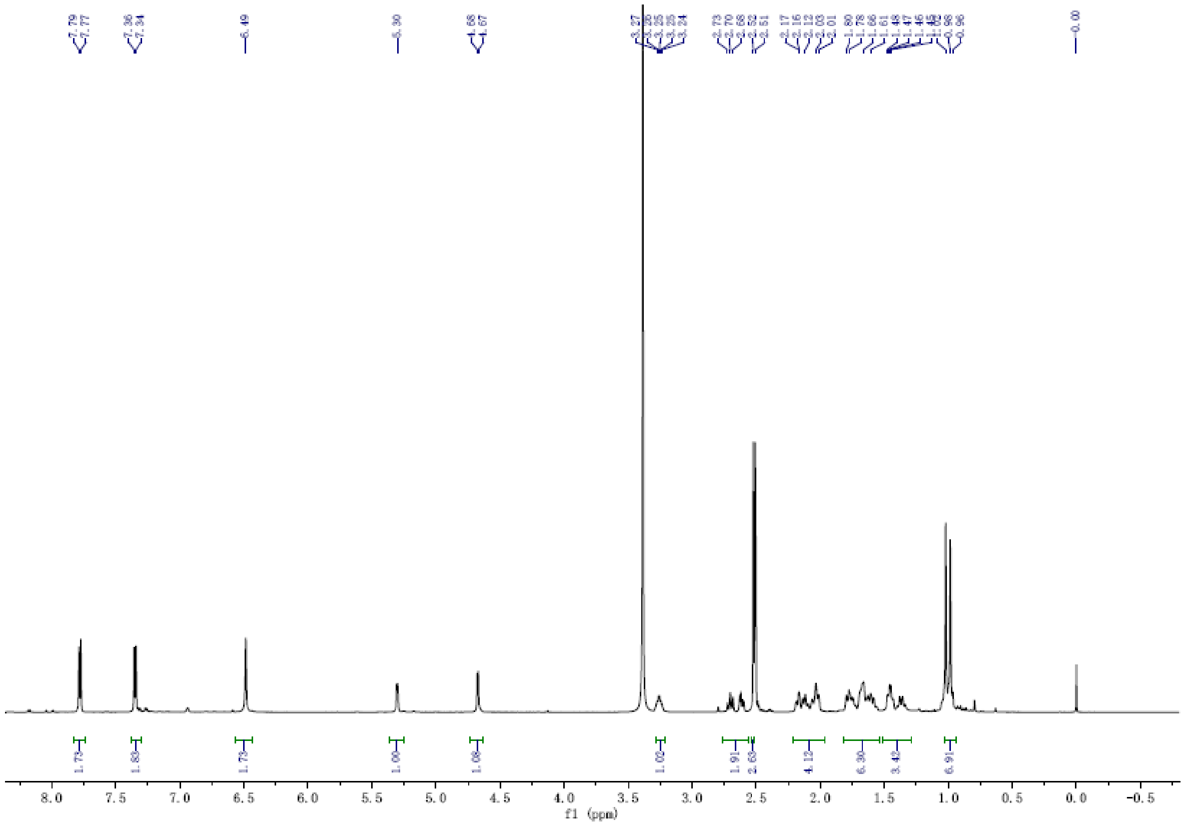

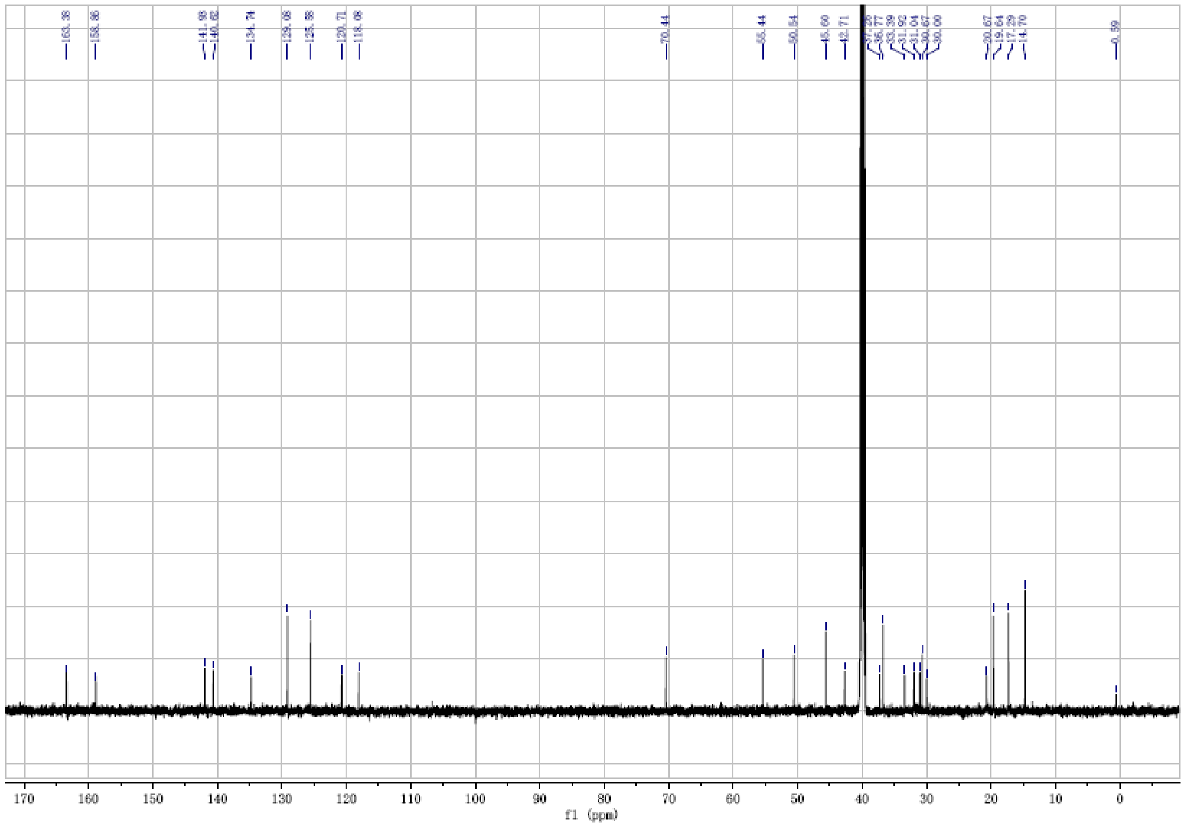

Compd 3l

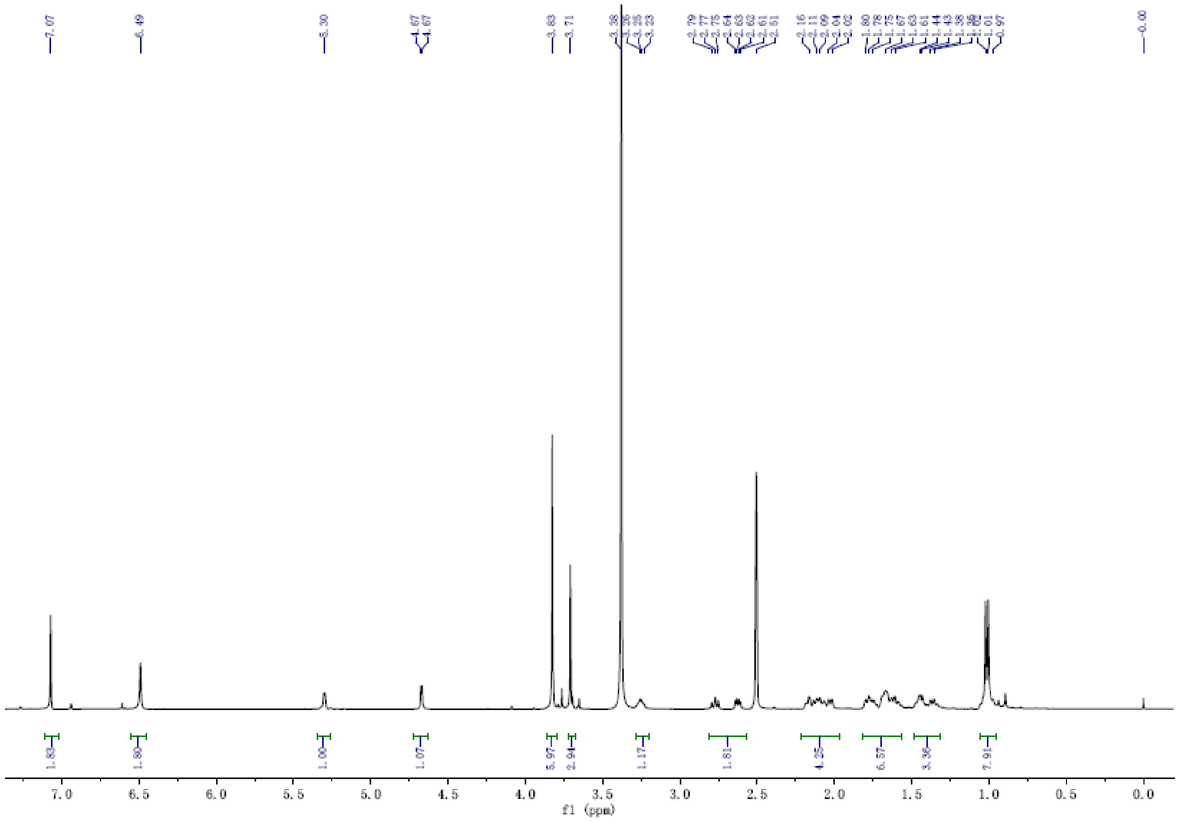

7.07
6.49
5.30
4.67
4.67
3.83
3.71
3.38
3.26
3.25
3.23
2.79
2.77
2.75
2.64
2.63
2.62
2.61
2.51
2.16
2.11
2.09
2.04
2.02
1.80
1.78
1.75
1.67
1.63
1.61
1.44
1.43
1.38
1.01
0.97
0.00
1.83
1.80
1.00
1.07
5.97
2.94
1.17
1.81
4.25
6.57
3.36
7.91
7.0
6.5
6.0
5.5
5.0
4.5
4.0
3.5
3.0
2.5
2.0
1.5
1.0
0.5
0.0
f1 (ppm)

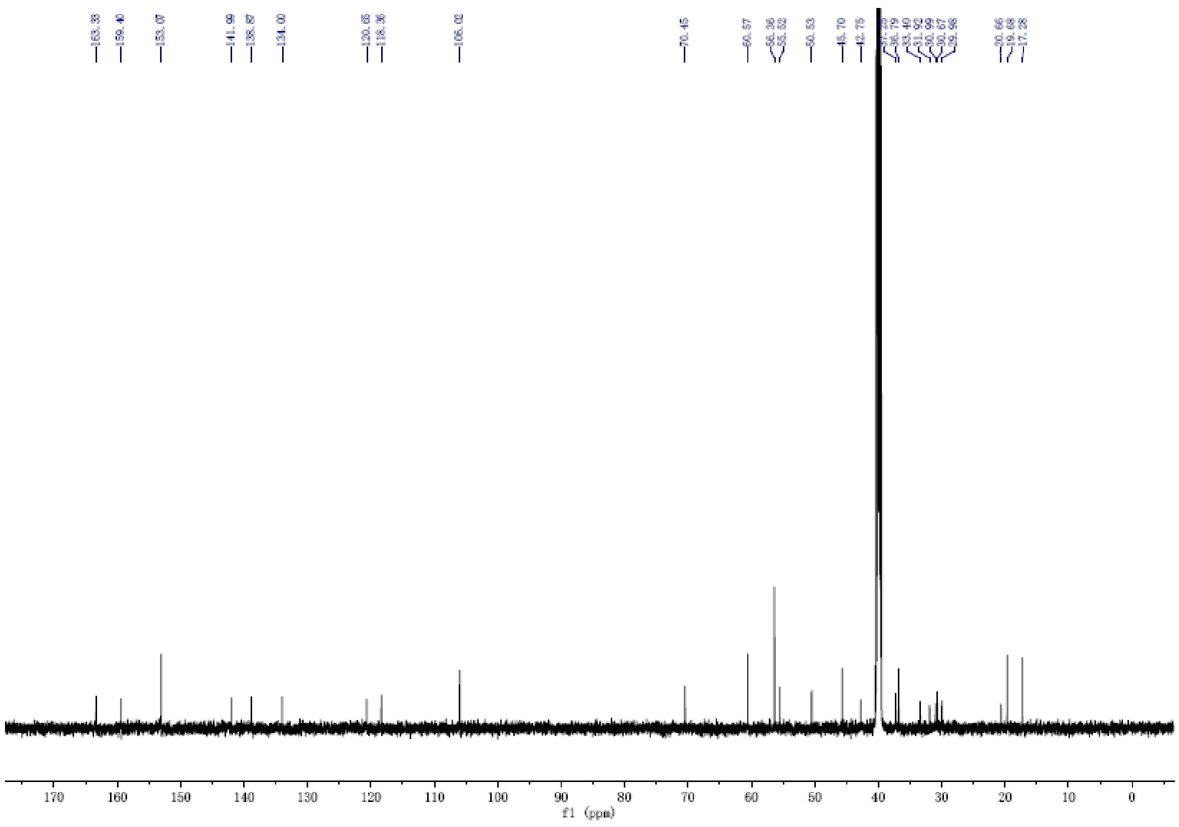

163.33
159.40
153.07
141.99
138.87
134.00
120.65
118.36
106.02
70.45
60.57
56.36
55.52
50.53
45.70
42.75
36.79
33.40
31.92
30.99
30.67
29.58
20.66
19.68
17.28
170
160
150
140
130
120
110
100
90
80
70
60
50
40
30
20
10
0
f1 (ppm)

Compd 3m

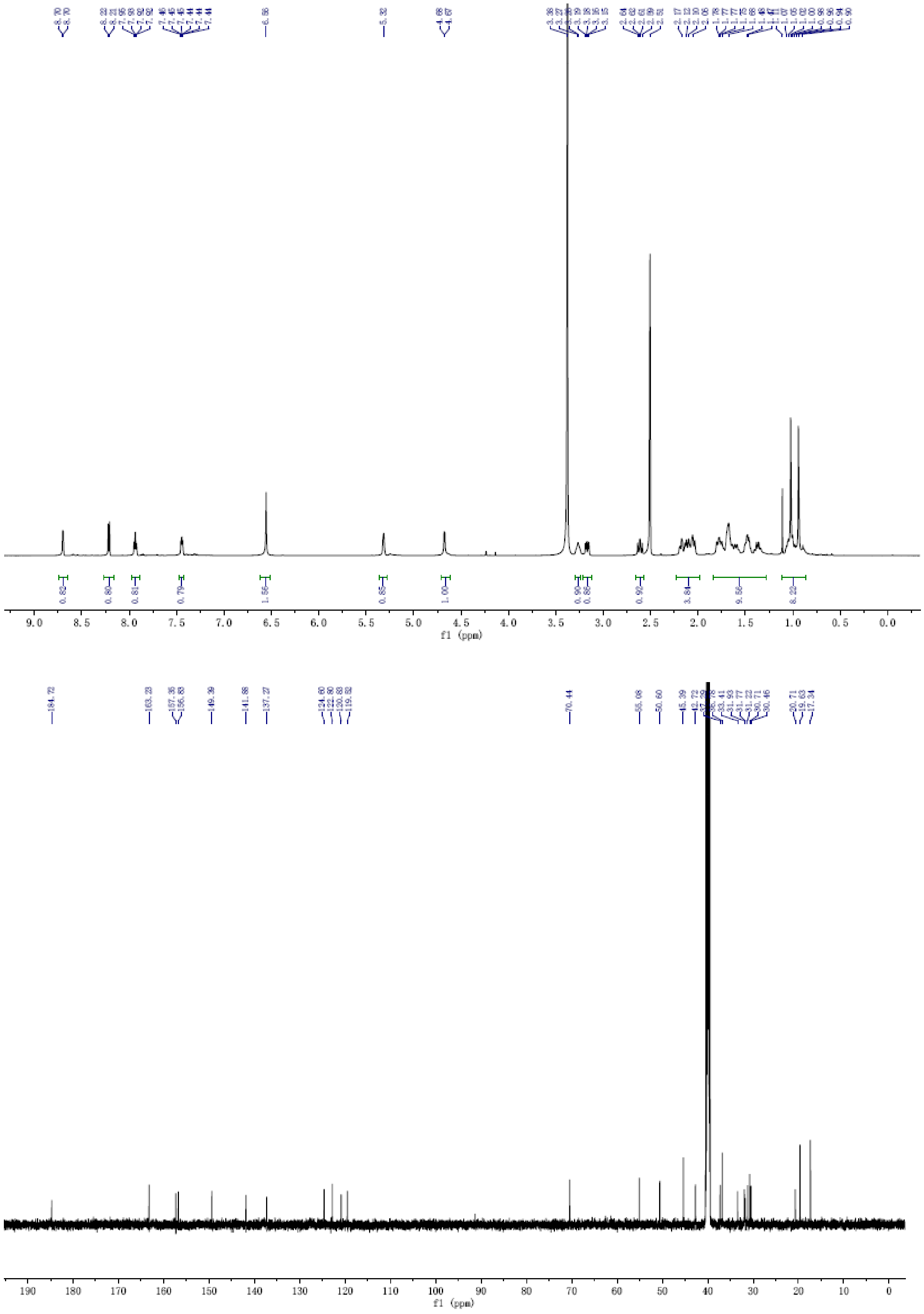

Compd 3n

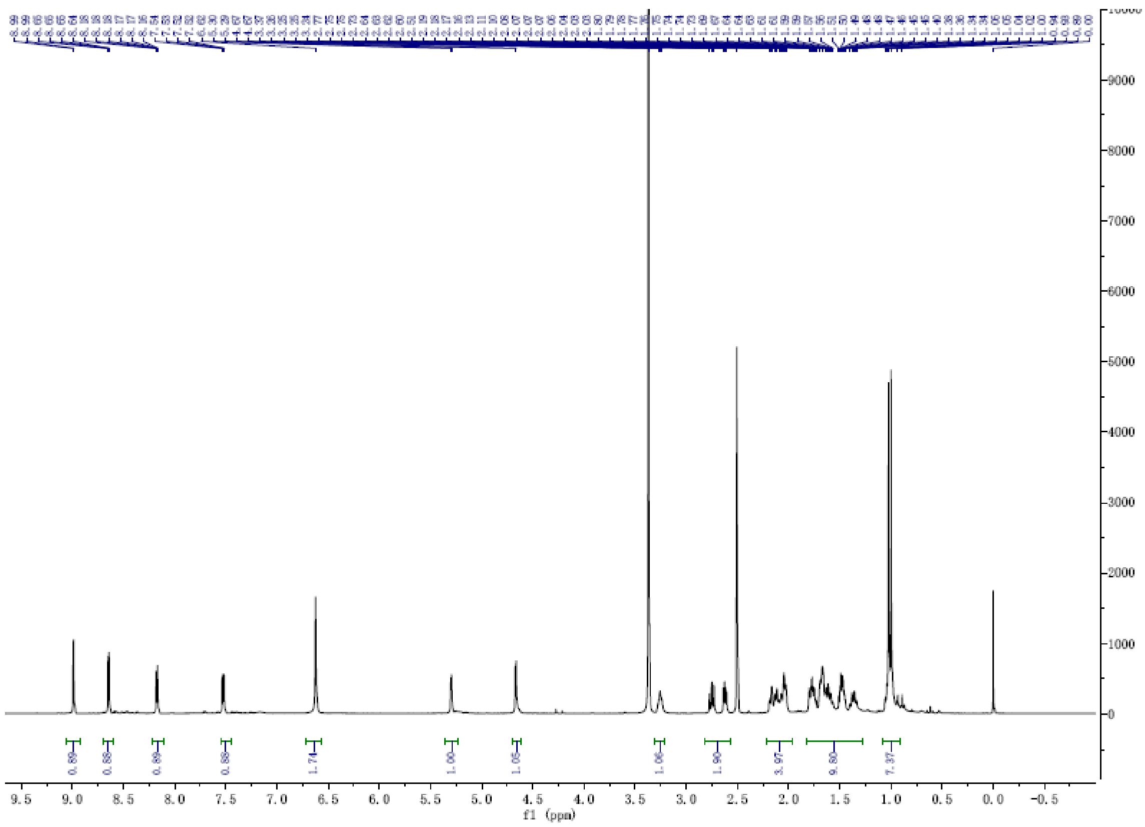

f1 (ppm)

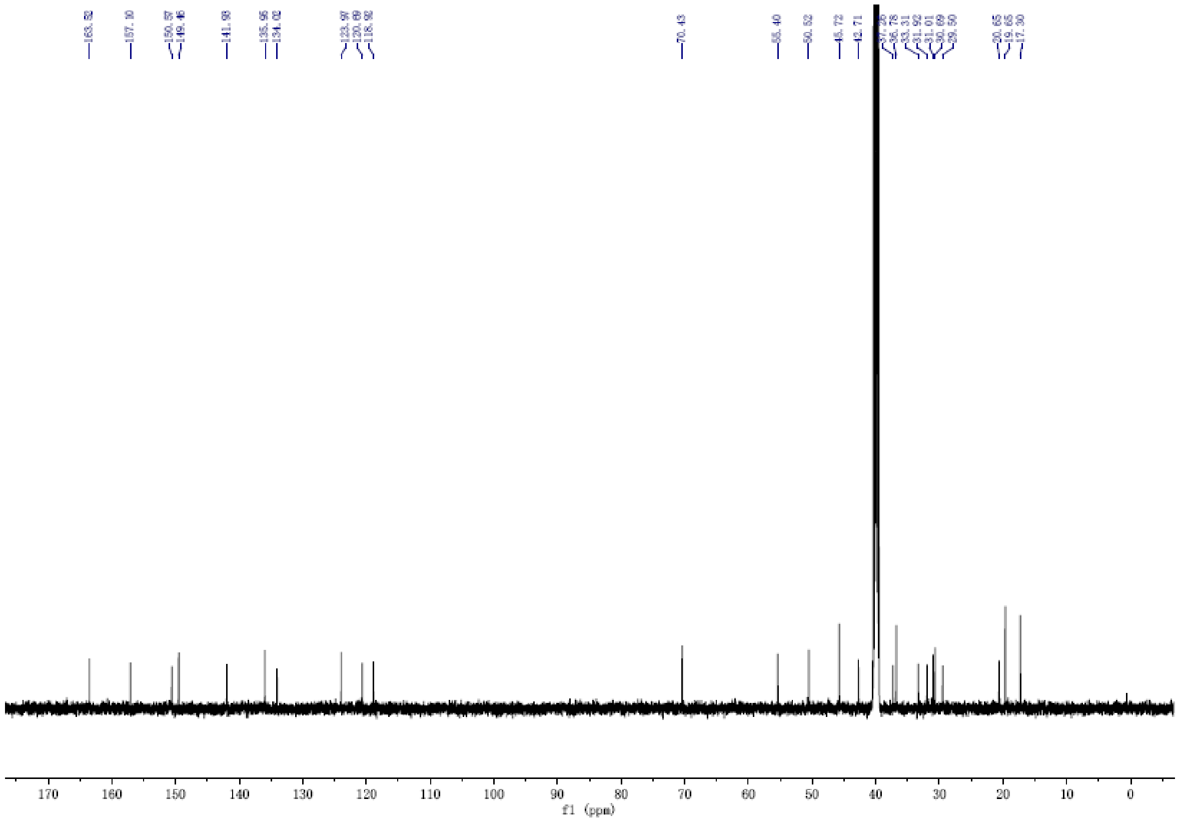

163.82
157.30
150.57
149.46
141.98
135.96
134.02
123.97
120.69
118.92
70.43
55.40
50.52
45.72
42.71
37.26
36.78
33.31
31.92
31.01
30.69
29.50
20.65
19.65
17.30
f1 (ppm)

Compd 3o

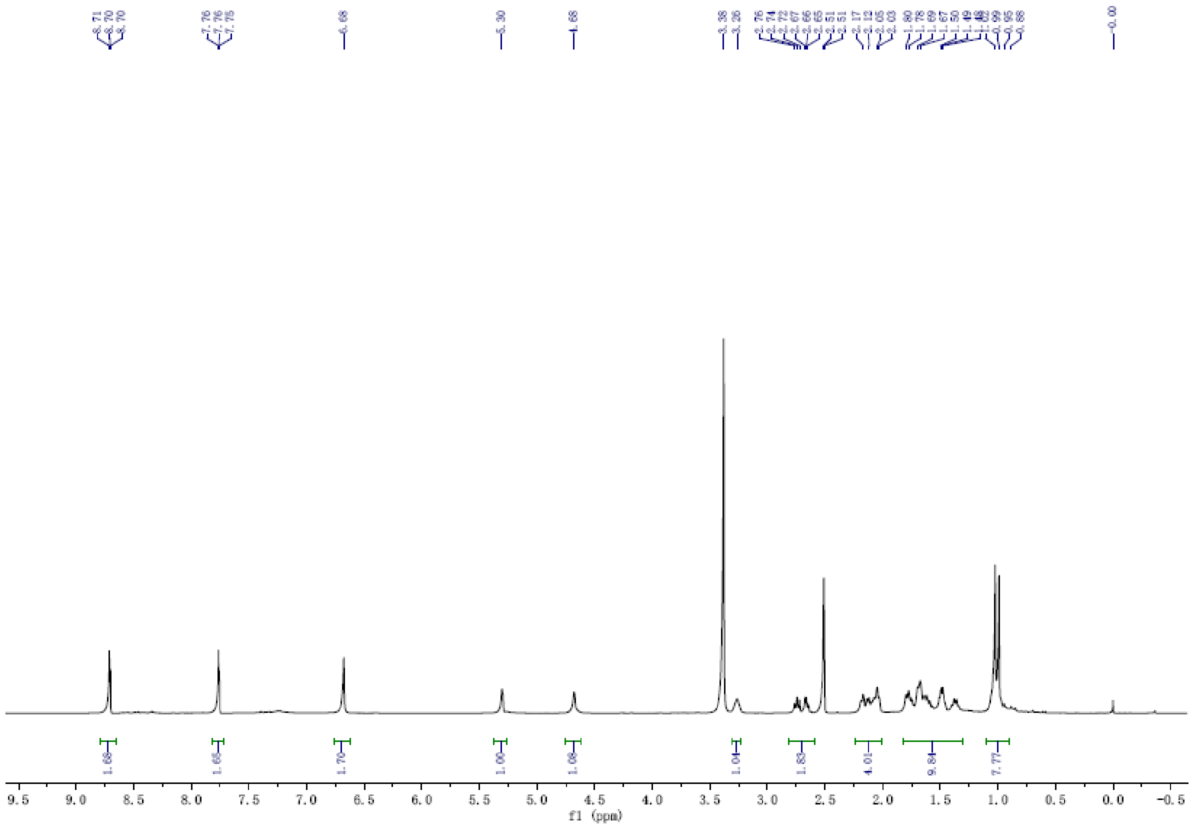

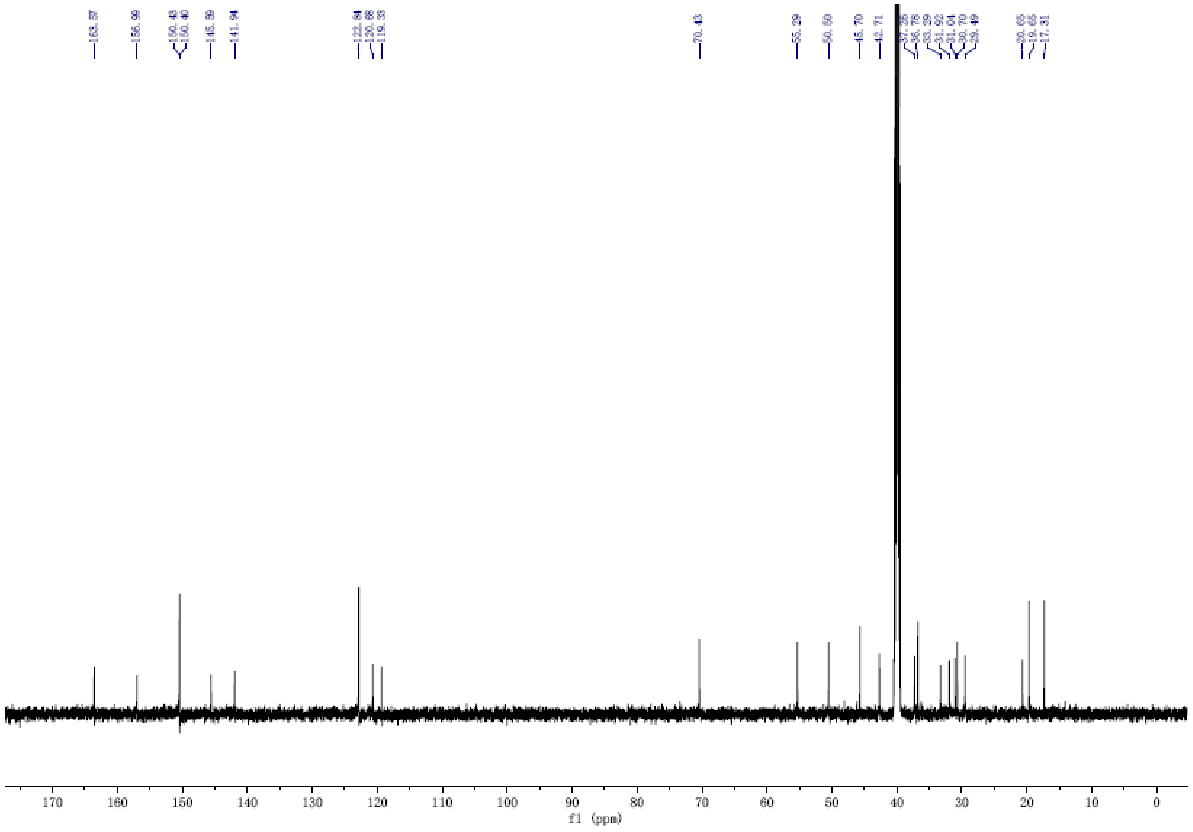

Compd 3p

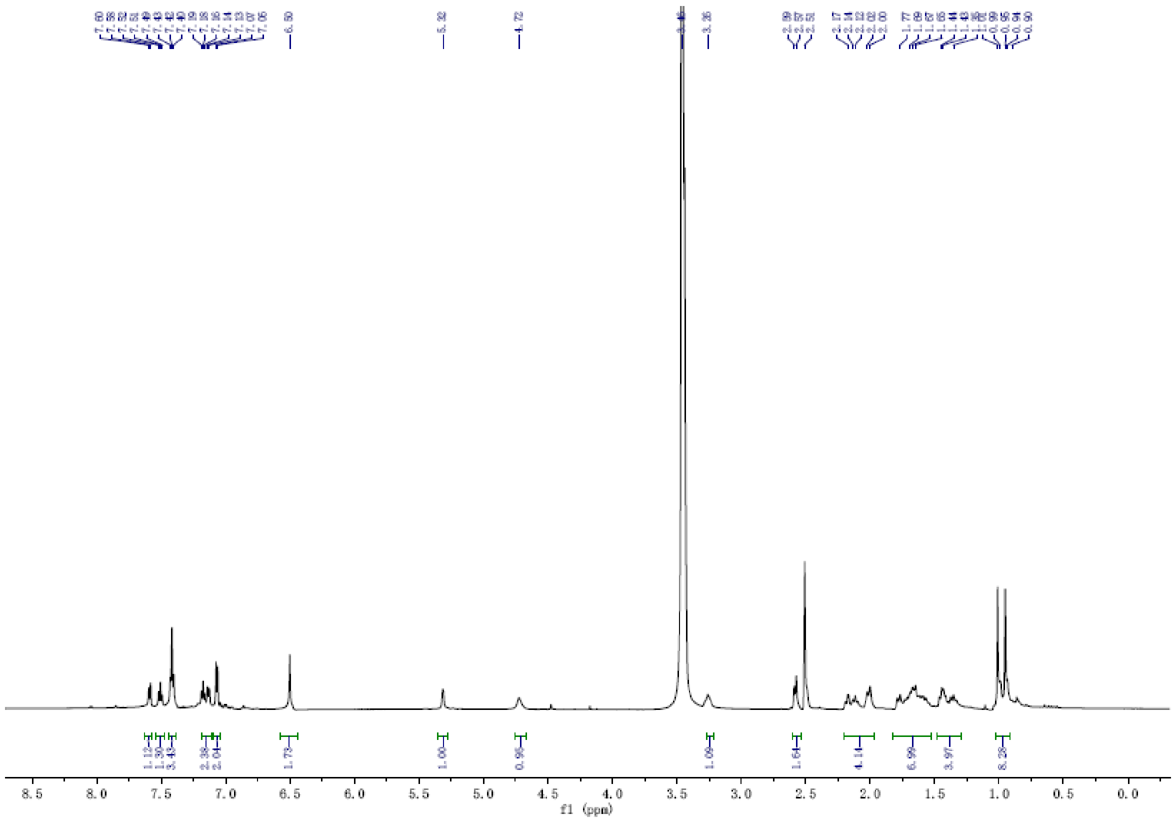

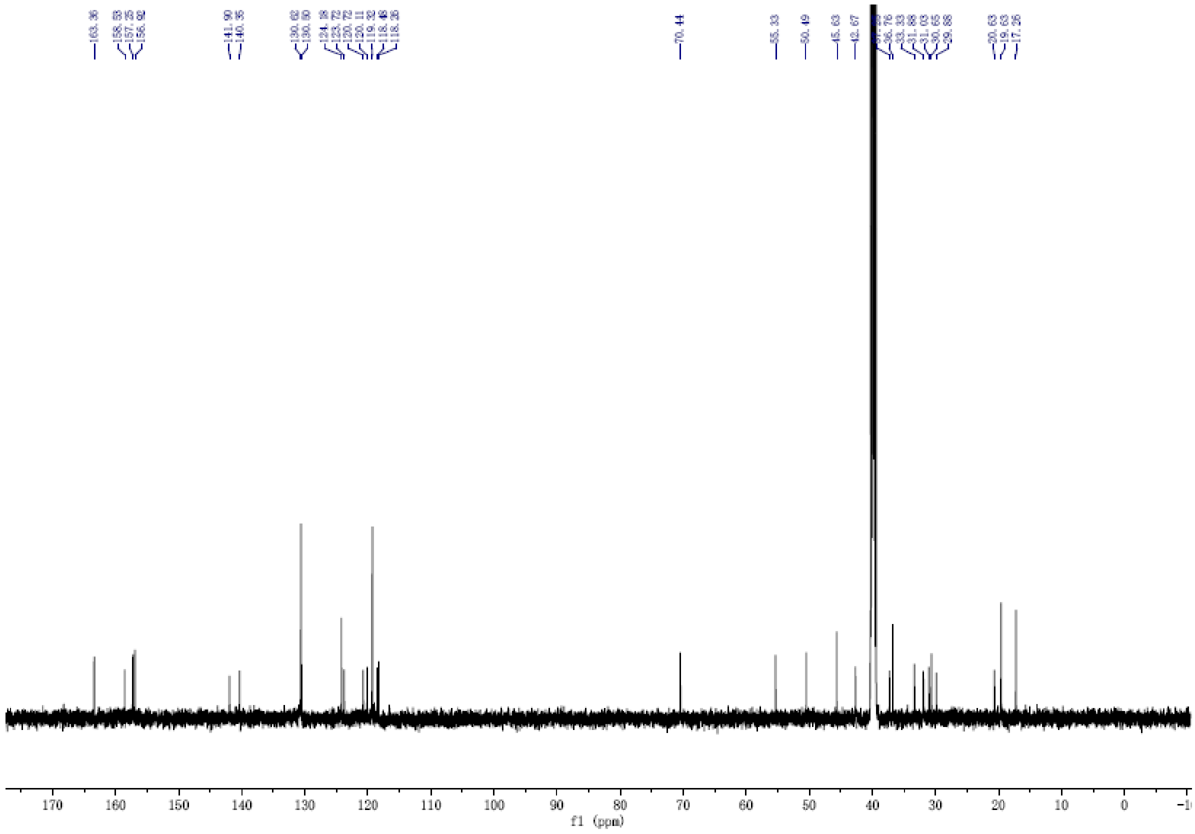

Supplement: Supporting Information [file srep44439-s1.pdf]
